# Characterizing and Predicting Post-Acute Sequelae of SARS CoV-2 Infection (PASC) in a Large Academic Medical Center in the US

**DOI:** 10.3390/jcm12041328

**Published:** 2023-02-07

**Authors:** Lars G. Fritsche, Weijia Jin, Andrew J. Admon, Bhramar Mukherjee

**Affiliations:** 1 Department of Biostatistics, University of Michigan School of Public Health, Ann Arbor, MI 48109, USA; 2 Center for Precision Health Data Science, University of Michigan School of Public Health, Ann Arbor, MI 48109, USA; 3 Division of Pulmonary and Critical Care Medicine, Department of Internal Medicine, University of Michigan Medical School, Ann Arbor, MI 48109, USA; 4 Department of Epidemiology, University of Michigan School of Public Health, Ann Arbor, MI 48109, USA; 5 VA Center for Clinical Management Research, LTC Charles S. Kettles VA Medical Center, Ann Arbor, MI 48109, USA; 6 Michigan Institute for Data Science, University of Michigan, Ann Arbor, MI 48109, USA

**Keywords:** Coronavirus Disease-2019 (COVID-19), post-acute sequelae of SARS CoV-2 (PASC, long COVID, post-COVID conditions), phenome-wide association study, phenotype risk score, electronic health records

## Abstract

Background: A growing number of Coronavirus Disease-2019 (COVID-19) survivors are affected by post-acute sequelae of SARS CoV-2 infection (PACS). Using electronic health record data, we aimed to characterize PASC-associated diagnoses and develop risk prediction models. Methods: In our cohort of 63,675 patients with a history of COVID-19, 1724 (2.7%) had a recorded PASC diagnosis. We used a case–control study design and phenome-wide scans to characterize PASC-associated phenotypes of the pre-, acute-, and post-COVID-19 periods. We also integrated PASC-associated phenotypes into phenotype risk scores (PheRSs) and evaluated their predictive performance. Results: In the post-COVID-19 period, known PASC symptoms (e.g., shortness of breath, malaise/fatigue) and musculoskeletal, infectious, and digestive disorders were enriched among PASC cases. We found seven phenotypes in the pre-COVID-19 period (e.g., irritable bowel syndrome, concussion, nausea/vomiting) and sixty-nine phenotypes in the acute-COVID-19 period (predominantly respiratory, circulatory, neurological) associated with PASC. The derived pre- and acute-COVID-19 PheRSs stratified risk well, e.g., the combined PheRSs identified a quarter of the cohort with a history of COVID-19 with a 3.5-fold increased risk (95% CI: 2.19, 5.55) for PASC compared to the bottom 50%. Conclusions: The uncovered PASC-associated diagnoses across categories highlighted a complex arrangement of presenting and likely predisposing features, some with potential for risk stratification approaches.

## 1. Introduction

Coronavirus Disease-2019 (COVID-19) has posed unprecedented challenges to the public health and healthcare system. As of 30 September 2022, 96,158,524 confirmed COVID-19 cases were in the US [1]. Studies suggest that 20 to 40% of patients with a history of COVID-19 may be affected by post-acute sequelae of COVID-19 (PASC) [2,3,4]—also termed post COVID conditions (PCC), [5,6], long COVID [7], post-acute COVID-19 syndrome (PACS) [8], chronic COVID-19 syndrome [9], and long haul COVID-19 [10]. PASC is an aggregate term for a highly heterogeneous group of post-COVID-19 problems, including persistent symptoms of acute infection (e.g., cough, fatigue, loss of smell [11,12,13]), new chronic disorders, (e.g., chronic lung or neurologic disease [3,14,15,16,17,18,19,20,21]), and late post-COVID complications (e.g., autoimmune complications). COVID-19 vaccinations could decrease the risk for PASC by 13%–22% [22,23]; however, with a massive number of breakthrough infections and a relaxation of mitigation measures throughout the world, the high prevalence of PASC during an ongoing pandemic could present a tremendous burden for healthcare systems worldwide.

Several demographic factors, preexisting conditions, and biomarkers have been associated with PASC. For example, severe acute COVID-19, female gender, older age, pre-existing diabetes, or the experience of specific symptoms during the acute COVID-19 phase, including fatigue, headache, hoarse voice, etc., were reported to increase the risk for PASC [24,25,26,27]. A previous investigation reported an immunoglobulin (Ig) signature, based on total IgM and IgG3, as a predictor for PASC [28], while another study identified a series of features, including the rate of healthcare utilization, patient age, dyspnea, and other diagnosis and medication information, to predict PASC [29]. Another study identified four risk factors: type 2 diabetes, the presence of SARS-CoV-2 RNA, Epstein–Barr virus, and specific auto-antibodies [30]. Together, these studies highlight the possibility and the need to uncover and understand PASC risk factors to identify and protect vulnerable groups. Furthermore, a better understanding of PASC might allow the identification of PASC subtypes and their specific risk profiles. However, the novelty of this condition and the sparsity of studies so far have hampered the development of risk-prediction models for PASC.

In our current study, we aim to fill this gap by identifying predisposing diagnoses of PASC through phenome-wide association studies (PheWAS) of the pre-COVID-19 and acute-COVID-19 time periods and then use the identified pre-existing conditions to develop and evaluate integrated and usable phenotype risk scores (PheRS) [31] to predict PASC [32,33]. To do this, we leverage a cohort of over 60,000 patients with a history of COVID-19 cared for at Michigan Medicine (MM), a large academic medical center in the Midwestern US, between March 2020 and August 2022. This cohort includes 1724 patients that were subsequently diagnosed with PASC using diagnostic codes or clinical problem lists. With its rich retrospective EHR data that includes socioeconomic status (SES), demographics, and other relevant variables, this cohort offers a unique opportunity to study PASC.

## 2. Materials and Methods

### 2.1. Study Cohort

The study included Michigan Medicine (MM) patients with a recorded COVID-19 diagnosis or a positive real-time reverse transcriptase chain (RT-PCR) test for SARS-CoV-2 infection performed/recorded at MM between 10 March 2020, and 31 August 2022. Diagnoses were recorded at clinic visits and hospital encounters. RT-PCR testing data were collected for routine screening at hospital admission, before procedures, and for employee screening. Tests included both symptomatic and asymptomatic individuals.

For each subject, the date of their first COVID-19 diagnosis or RT-PCR positive test, whichever came first, was considered the index date. Dates were regarded as protected health information and operationalized as days since birth; however, the quarter of the year of the index date was obtained. To allow sufficient follow-up time for diagnosing PASC, we limited the analysis to patients with encounters at least two months after being COVID-19 positive and stratified them in PASC cases (had a recorded PASC diagnosis) and PASC controls (had no recorded PASC diagnosis).

**Figure 1:**
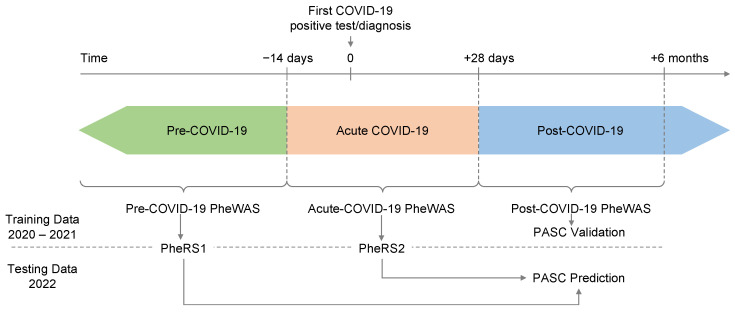
Schematic on study design. Three time periods were defined relative to the 1. positive COVID-19 test or diagnosis (index date): pre-COVID-19 until −14 days, acute-COVID-19 from −14 to +28 days, and post-COVID-19 from +28 days onwards. The post-COVID-19 PheWAS is used to validate features of PASC cases compared to COVID-19 cases without PASC diagnoses. The pre-COVID-19 and acute-COVID-19 PheWAS on the training data (index date in 2020–2021) inform on phenotype risk scores (PheRS) that will be used to predict PASC in the testing data (index date in 2022).

PASC diagnoses were either based on an entry of PASC in the diagnosis section of the EHR database’s Problem Summary List (PSL, Table S1) or on observations of the ICD-10-CM (International Classification of Diseases codes, tenth edition with clinical modifications) U09.9 (“Post COVID-19 condition, unspecified”) or B94.8 (“Sequelae of other specified infectious and parasitic diseases”). The CDC recommended the latter as a temporary alternative to the PASC-specific U09.9 code, which was implemented on 1 October 2021 [34]. PSL diagnoses represent active and resolved patient problems entered by healthcare providers. The age at the first observed ICD- or PSL-based PASC diagnosis was considered the age of onset of PASC. PASC cases (see definition below) without a prior positive test were excluded because the timepoint of the test was crucial for defining the pre-COVID-19 and acute-COVID-19 time periods (Figure 1).

We also categorized PASC patients based on ICD10 diagnoses concurrently recorded with their first PASC diagnosis and mapped them to 29 phenotype concepts previously reported as common PASC symptoms [3]. In addition, we manually mapped detailed PSL diagnoses to these 29 concepts (Tables S1 and S2).

### 2.2. Definition of Demographics, Socioeconomic Status, and Other Covariates

To examine and adjust for confounding by patient characteristics, socioeconomic status, and other variables, we obtained the following data for each participant: age, self-reported gender, self-reported race/ethnicity, neighborhood disadvantage index (NDI) without proportion of Black (coded as quartiles, with larger quartiles representing more disadvantaged communities) [35,36], and population density measured in persons per square mile (operationalized as quartiles).

Additional covariates included vaccination status, the Elixhauser comorbidity score [37,38], COVID-19 severity (non-severe (not hospitalized) and severe (hospitalized or deceased)), healthcare worker (HCW) status, the timespan of records in the EHR before and after the COVID-19 test/diagnosis, the timespan of records in the EHR before 2020 (referred to as “pre-pandemic” time period). These timespans were based on the first or last recorded encounter in the EHR data. Additional details and definitions of these covariates can be found in Appendix A and Table S3.

We assumed completely at random missingness of the covariates included in our adjusted analyses and performed complete case analyses for each adjustment.

### 2.3. Time-Restricted Phenomes

We constructed each subject’s medical phenome by extracting available ICD9 and ICD10 codes from the EHR and mapping them to 1813 broader phenotype concepts (PheCodes) using the R package “PheWAS” [39,40]. In short, individuals with ICD codes that map to a specific PheCode were coded as “1”, then individuals with ICD codes that map to the PheCode’s specific exclusion criteria were coded as missing, and finally, all remaining individuals were coded as “0” for that particular PheCode (further details are described elsewhere [40]). We created three time-restricted phenomes relative to the index date: post-COVID-19 (+28 days to +6 months), pre-COVID-19 (predating −2 weeks), and acute COVID-19 (−14 and +28 days; Figure 1).

### 2.4. Matching

To minimize confounding when we compare PASC (case) versus no PASC (control), we matched each PASC case to up to 10 PASC controls using the R package “MatchIt” [41]. Nearest neighbor covariate matching was applied for age at index date, pre-COVID-19 years in EHR, and post-COVID-19 years in EHR without applying a caliper. Exact matching was used for sex, primary care visit at Michigan Medicine within the last two years (yes/no), race/ethnicity, and year quarter of the index date. We retained the case–control matching throughout all analyses.

### 2.5. Statistical Analysis

#### 2.5.1. PASC-Associated PheCodes in Post COVID-19 Period

To characterize diagnoses enriched in COVID-19 patients with PASC, we also conducted PheWAS to identify phenotypes associated with PASC in the post-COVID-19 period (at least 28 days after the COVID-19 index date, see Figure 1) using Firth bias-corrected logistic regression by fitting the following model for each PheCode of the post-COVID-19 period phenome:

(1) $$\begin{array}{ll} logit\;(P(PheCode & =1\;|\;PASC,\;Covariates))\\ & =\beta_{0}+\beta_{PASC}\mathrm{PASC}+\beta_{Covariate\;1}\;\mathrm{Covariate}\;1+\beta_{Covariate\;2}\;\mathrm{Covariate}\;2\\ & +\ldots +\beta_{Covariate\;p}\;\mathrm{Covariate}\;p \end{array}$$

where covariates were pre-COVID-19 Elixhauser Score (AHRQ), NDI, population density, healthcare worker status (HCW), vaccination status, and severity, details are summarized in Appendix A and Table S3.

#### 2.5.2. Pre-Disposing PheCodes

We conducted PheWAS to identify PheCodes pre-disposing to PASC using either PheCodes from the pre-COVID-19 period or PheCodes from the acute-COVID-19 period. We ran Firth bias-corrected logistic regression by fitting the following model for each PheCode of the corresponding time-restricted phenome:

(2) $$\begin{array}{ll} logit\;(P(PASC & =1\;|\;Phecode\;is\;present,\;Covariates))\\ & =\beta_{0}+\beta_{PheCODE}\mathrm{PheCODE}+\beta_{Covariate\;1}\;\mathrm{Covariate}\;1\\ & +\beta_{Covariate\;2}\;\mathrm{Covariate}\;2+...+\beta_{Covariate\;p}\;\mathrm{Covariate}\;p \end{array}$$

We applied a similar set of covariate adjustments as before (Table S3).

The phenomes were split into a training set (index dates in 2020 and 2021) and a testing set (index date in 2022). This choice was to retain the true spirit of future prediction using past data. The training set was used to identify predisposing PheCodes in phenome-wide association studies (PheWAS), while the testing set was used to evaluate prediction models based on the PheWAS results.

To evaluate the robustness of effect sizes of predisposing PheCodes, we performed several sensitivity analyses: (1) females only, (2) males only, (3) index date in 2020, (4) index date in 2021, (5) non-severe outcomes (not hospitalized), (6) severe outcomes (hospitalized or deceased), (7) recorded within two years before the index date, and (8) pre-pandemic (before 2020). For the acute-COVID-19 PheWAS, we excluded PASC cases whose first recorded PASC diagnosis was observed less than 28 days after the index date. The sample sizes of the complete case analyses for various analyses are listed in Table S4.

PheWASs were restricted to PheCodes observed at least five times among cases and among controls. For all PheWAS, we excluded PheCode 136 “Other infectious and parasitic diseases” as it included the ICD-10 code “B94.8” which was used to record a PASC diagnosis.

For each PheWAS, we applied a Bonferroni correction adjusting for the number of analyzed PheCodes (Table S4). In Manhattan plots, we present –log10 (*p*-value) corresponding to tests for association of the underlying phenotype. Directional triangles on the PheWAS plot indicate whether a trait was positively (pointing up) or negatively (pointing down) associated.

We also tested for differences between effect sizes of three subgroup comparisons (non-severe vs. severe outcome, female vs. male, and index date in 2020 vs. 2021) using the following t-statistics:

(3) $$t=\frac{\beta_{A}-\beta_{B}}{\sqrt{SE{\left(\beta_{A}\right)}^{2}+SE{\left(\beta_{B}\right)}^{2}}}$$

where $\beta_{A}$ and $\beta_{B}$ are the subgroup-specific beta-estimates with corresponding standard errors $SE\left(\beta_{A}\right)$ and $SE\left(\beta_{B}\right)$.

#### 2.5.3. Phenotype Risk Scores (PheRS)

##### PheRS Generation

To generate the phenotype risk score or PheRS, we first screened the PheWAS for PheCodes that were phenome-wide significant at a Bonferroni corrected threshold in a one-at-a-time analysis in terms of their association with PASC (after adjusting for covariates). Next, we ran a joint multivariate model with all phenome-wide significant PheCodes using ridge penalized logistic regression (R Package “glmnet” [42,43]) to obtain the adjusted coefficients/weights per PheCode from the training data before calculating the PheRS in the testing data. More specifically, we weighted the presence of PheCodes with their adjusted coefficients from the multivariate ridge penalized logistic regression and calculated the PheRS as the weighted sum. For subject *j*, the PheRS was of the form PheRS*_j_* = $\sum_{i}\hat{\beta_{i}\;}\;PheCode_{ij}$ where the sum extends over all included PheCodes, ${\hat{\beta }}_{i}$ are the adjusted ridge regression coefficients for PheCode *i* from the multivariate model, and $PheCode_{ij}$ denotes the presence/absence (coded as 1 and 0) of a PheCode *i* in subject *j.* We used Ridge regression because it has been shown to offer good performance when there is multicollinearity between features, and when prediction is the goal [44].

##### PheRS Evaluation

To evaluate each of the PheRS, we fit the following Firth bias-corrected logistic regression model adjusting for age, gender, race/ethnicity, Elixhauser Score, population density, NDI, HCW, vaccination status, pre-COVID19 years in EHR and severity using a complete case analysis:

(4) $$\begin{array}{ll} logit\;(P(PASC & =1\;|\;PheRS,\;Covariates))\\ & =\beta_{0}+\beta_{PheRS}\mathrm{PheRS}+\beta_{Covariate\;1}\mathrm{Covariate}\;1\\ & +\beta_{Covariate\;2}\;\mathrm{Covariate}\;2+...+\beta_{Covariate\;p}\mathrm{Covariate}\;p \end{array}$$

For each PheRS, we assessed the following performance measures relative to the PASC status: (1) overall performance with Nagelkerke’s pseudo-R^2^ using R packages “rcompanion” [45], (2) accuracy with Brier score using R package “DescTools” [46]; and (3) ability to discriminate between PASC cases and matched controls as measured by the area under the covariate-adjusted receiver operating characteristic (AROC; semiparametric frequentist inference) curve (denoted AAUC) using R package “ROCnReg” [47]. Firth’s bias reduction method was used to resolve the problem of separation in logistic regression (R package “brglm2”) [48].

To also evaluate models with both predictors (PheRS1-Ridge + PheRS2-Ridge), we combined them by first fitting a logistic regression with the predictors in the training set to obtain the linear predictors that we used to obtain the combined score in the testing data.

Unless otherwise stated, analyses were performed using R 4.2.0 [49].

## 3. Results

### 3.1. Patient Characteristics

Among 63,675 patients with a history of COVID-19 who were seen in MM at least two months after their first record of COVID-19, 1724 (2.7%) received a PASC diagnosis. The PASC prevalence within three months of a COVID-19 infection ranged from 0.18% (Q3 of 2020) to 1.8% (Q3 of 2021). The most PASC cases were observed in Q4/2021 (*n* = 134), coinciding with the second peak of positive tests at MM (Table 1; Figure S1).

We observed that PASC cases compared to controls were on average older at their index date (mean age 47.9 versus 41.7 years), had a slightly longer timespan covered in the pre-test EHRs (11.7 versus 10.4 years), were more likely female (64.5% versus 56.7%), more likely to have received primary care at MM in the last two years (60.7% versus 46.4%) and showed different distributions across the year quarters over time (Table 1). To adjust for these observed differences, we performed nearest neighbor matching (age at index date, pre-test years in EHR, post-test years in EHR) and exact matching (gender, primary care at MM, race/ethnicity, quarter of year at COVID-19 index date). All significant differences in covariates became non-significant after matching (Table 1).

### 3.2. PASC Symptoms/Post-COVID-19 PheWAS

When categorizing 1362 PASC cases with concurrent diagnoses based on 29 previously reported symptoms [3] (362 of the 1724 cases had no concurrent diagnoses, Tables S1 and S2), the 10 most common diagnoses were: shortness of breath (34.3%), anxiety (30.6%), malaise and fatigue (28.5%), depression (27.2%), sleep disorders (25.4%), asthma (23.6%), headaches (21.4%), migraine (13.8%), cough (13.0%) and joint pain (12.6%) (Table S5).

**Figure 2:**
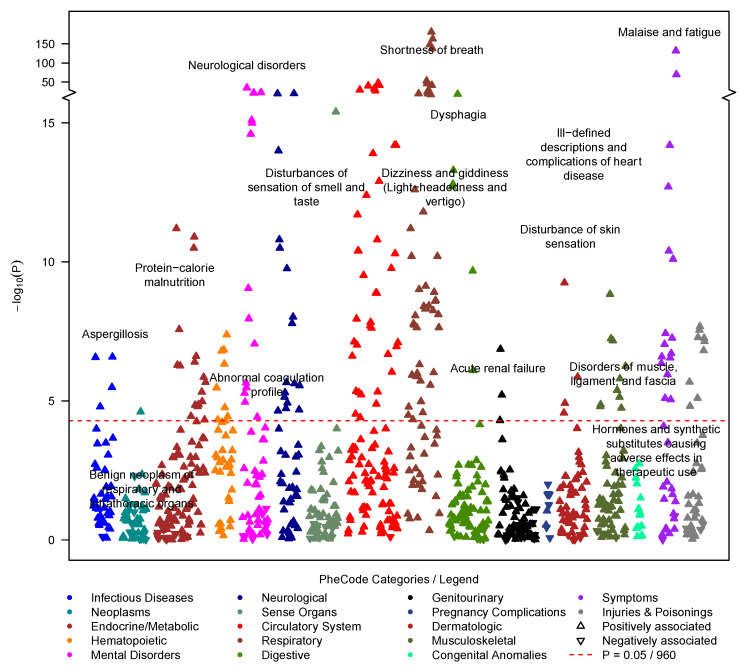
PheWAS on symptoms that occurred between 28 days and 6 months after the first COVID-19 test (outcome: post-COVID-19 symptoms/phecodes; predictor: PASC diagnosis yes/no). Among PheCodes that reached phenome-wide significance (red dashed line, *p* ≤ 0.05/960 = 5.2 × 10^−5^), only the strongest association per PheCode category was labeled. The analysis was adjusted using the following covariates: age at index date, gender, race/ethnicity, Elixhauser Score AHRQ, population density (quartiles), NDI (quartiles), health care worker status, vaccination status, post-test years in EHR, and severity. Summary statistics can be found in File S1.

In the post-COVID-19 PheWAS of 1256 cases versus 12,492 matched controls, all 29 PASC symptoms were enriched among PASC cases (OR > 1), and 27 reached phenome-wide significance (*p* < 0.05/960 tested PheCodes; *p* < 5.2 × 10^−5^) while 2 were not significant (Table S6). In addition to PASC-related phenotypes (e.g., shortness of breath: OR = 9.03 [7.77, 10.50], *p* = 2.94 × 10^−181^; malaise and fatigue: OR = 6.17 [5.33, 7.14], *p* = 2.32 × 10^−132^; and cardiac dysrhythmias: OR = 2.75 [2.37, 3.18], *p* = 3.95 × 10^−41^), many additional diagnoses were enriched in PASC cases, among others musculoskeletal disorders (e.g., costochondritis: OR = 6.88 [95%: 3.05, 14.8], *p* = 6.72 × 10^−8^), infectious diseases (e.g., septicemia: OR = 2.31 [1.66, 3.16] *p* = 2.67 × 10^−7^), and digestive disorders (e.g., gastroesophageal reflux disease (GERD): OR = 1.72 [1.50, 1.99], *p* = 5.10 × 10^−14^) (Figure 2, File S1A).

### 3.3. Pre-COVID-19 PheWAS

**Figure 3:**
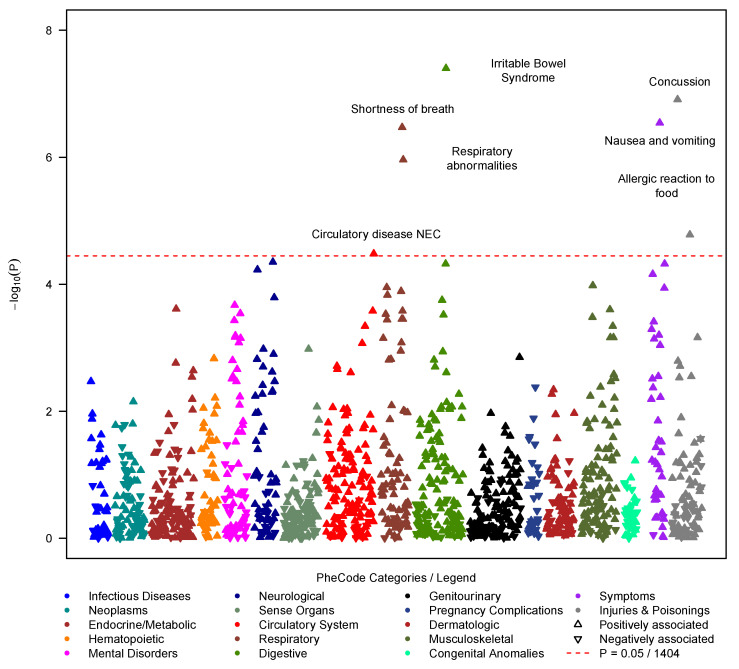
PheWAS on symptoms that occurred at least 14 days before the first positive COVID-19 test (outcome: PASC diagnosis yes/no; predictors: PheCodes). Among PheCodes that reached phenome-wide significance (red dashed line, *p* ≤ 0.05/1404 = 3.56 × 10^−5^), only the strongest association per PheCode category was labeled. The analysis was adjusted using the following covariates: age at index date, gender, race/ethnicity, Elixhauser Score, population density (quartiles), NDI (quartiles), health care worker status, vaccination status, pre-test years in EHR, and severity. Summary statistics can be found in File S1.

Of the 1724 individuals, 163 had incomplete covariate data. The 1561 remaining individuals were split into a training set (1212 individuals whose 1. positive test/diagnosis was recorded before 2022) and a testing set (349 individuals whose 1. positive test/diagnosis was recorded in 2022; also see flowchart in Figure S2). To identify potential PASC-predisposing conditions, we performed a PheWAS using the pre-COVID-19 phenome, comparing 1212 PASC cases versus 11,919 matched controls. Among 1405 tested PheCodes, 7 reached phenome-wide significance (*p* < 3.56 × 10^−5^): irritable bowel syndrome (IBS; OR = 1.78 [1.44, 2.18], *p* = 4.00 × 10^−8^), concussion (OR = 1.95 [1.51, 2.49], *p* = 1.24 × 10^−7^), nausea and vomiting (OR = 1.45 [1.26, 1.67], *p* = 2.90 × 10^−7^), shortness of breath (OR = 1.51 [1.29, 1.76] 3.38 × 10^−7^), respiratory abnormalities (OR = 1.39 [1.22, 1.59], *p* = 1.10 × 10^−6^), allergic reaction to food (OR = 1.94 [1.42, 2.60], *p* = 1.66 × 10^−5^) and general circulatory disease (OR = 1.52 [1.24, 1.85], *p* = 3.30 × 10^−5^; Figure 3, File S1B).

Additional sensitivity analyses indicated robust associations across various settings (females only, males only, 2020 only, 2021 only, non-severe outcome, severe outcomes, within two years before the index date, or before the pandemic, Figure S3A–G, File S1D–F).

### 3.4. Acute-COVID-19 PheWAS

**Figure 4:**
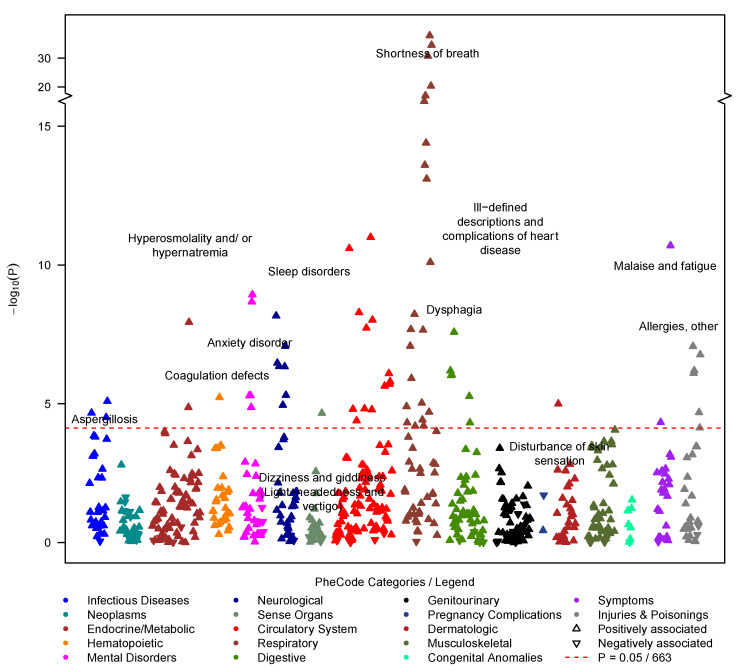
Acute-COVID-19 PheWAS on symptoms that occurred between −14 and +28 days relative to testing positive for COVID-19 (outcome: acute-COVID-19 symptoms/PheCodes; predictor: PASC diagnosis yes/no). Among PheCodes that reached phenome-wide significance (red dashed line, *p* ≤ 0.05/663 = 7.5 × 10^−5^), only the strongest association per PheCode category was labeled. The analysis was adjusted using the following covariates: age at index date, gender, race/ethnicity, Elixhauser Score AHRQ, population density (quartiles), NDI (quartiles), health care worker status, vaccination status, post-test years in EHR, and severity. Summary statistics can be found in File S1.

To uncover PASC-predisposing acute-COVID-19 symptoms, we screened 664 phenotypes of the acute-COVID-19 phenome, comparing 874 cases with 8671 controls. To not identify actual PASC symptoms compared to pre-PASC symptoms, we excluded cases whose PASC diagnosis was recorded less than 28 days after their index date and only retained their matched controls. A total of 69 phenotypes was significantly associated with PASC (*p* < 7.54 × 10^−5^) and included, among others, 22 respiratory phenotypes (e.g., shortness of breath, respiratory failure/insufficiency/arrest, dependence on a respirator or supplemental oxygen, and cough), 13 circulatory system phenotypes (orthostatic hypotension, hypotension), 7 neurological phenotypes (e.g., sleep disorder, migraine, pain), 6 digestive phenotypes (e.g., GERD, IBS), 5 mental health phenotypes (e.g., anxiety, depression), and other symptoms (e.g., malaise and fatigue, myalgia and myositis) (Figure 4, File S1C).

Our sensitivity analyses indicated robust associations across various settings (females only, males only, 2020 only, 2021 only, non-severe outcomes, severe outcomes) where most associations remained nominally significant in each sub-analyses or had overlapping confidence intervals in their sensitivity analyses. However, effect sizes were not as consistent (Figure S4A–AK, File S1G–I). Noteworthily, the effect size for shortness of breath differed significantly between index dates in 2020 and 2021 (2020: OR = 2.20 [1.60, 2.99], *p* = 7.8 × 10^−7^ compared to 2021: OR = 4.59 [3.62, 5.81], *p* = 9.37 × 10^−37^; P_Difference_ = 0.000234), though they were significantly associated with PASC in both years (Figure S4AA, File S1C,I). Despite low numbers of individuals with severe outcomes (160 PASC cases and 150 controls), 6 of the 69 significantly associated phenotypes (aspergillosis, bacterial pneumonia, MRSA pneumonia, hyperosmolality and/or hypernatremia, septic shock, and voice disturbances) only had sufficient observations among the subset with severe outcomes but among the non-severe outcome subset (724 PASC cases and 6799 controls; Table S4 and File S1C,G). This suggested that these phenotypes might be hospital-acquired complications. None of the 49 significantly associated phenotypes that were tested among individuals with non-severe outcomes and individuals with severe outcomes showed significant effect size differences (P_difference_ ≥ 0.001 [0.05/49 tests]). All phenotypes with nominal effect size differences between non-severe and severe outcomes (P_difference_ < 0.05) were all strongly and positively associated in individuals with non-severe outcomes, thus unlikely to merely represent hospital-acquired complications (File S1G).

### 3.5. Comparison of “Pre-PASC” Associated PheCode across Three PheWAS

To investigate whether the associated “pre-PASC” phenotypes of the pre- and acute-COVID-19 periods (“pre-PASC” phenotypes) are associated with novel PASC symptoms or if they become long-term features that manifest as PASC, we explored their frequencies and their association signals across all three PheWAS (Figure S5). Interestingly, almost all associated “pre-PASC” phenotypes were also significantly enriched in the post-COVID-19 PheWAS, except for “allergic reaction to food” of the pre-COVID-19 PheWAS and “candidiasis” and “inflammation and edema of the lung” in the acute-COVID-19 PheWAS. However, their ORs were all positive (File S1A–C). While we observed similarities between pre-existing conditions and presenting PASC features, further analyses using rigorous causal inference methods are needed to evaluate their causal role in developing PASC. The current analysis is merely correlative and a prediction exercise.

### 3.6. Developing Phenotype Risk Scores for Predicting PASC

The pre- and acute-COVID-19 PheWASs indicated pre-disposing conditions for PASC. To study whether these conditions might be helpful in predicting PASC among patients with a history of COVID-19, we generated two PheRSs: a pre-COVID-19 PheRS “PheRS1” and an acute-COVID-19 PheRS “PheRS2”. We avoided overfitting by using PheWAS results and PheRS weights obtained from individuals with index dates in 2020 or 2021, while the evaluations were performed in individuals with index dates in 2022 (Figure 1 and Figure S2 and File S1J). To limit the impact of potential hospital-acquired complications of an acute-COVID-19 infection, we excluded the six phenotypes that were only tested/observed in the individuals with severe outcomes (see “acute-COVID-19 PheWAS” above).

We found that PheRS1 and PheRS2 could discriminate cases and controls, yet only with low accuracy (AAUC < 0.7). PheRS1 performance was comparable in the complete testing data (AAUC_PheRS1_ = 0.548 [95% CI: 0.516, 0.580]) and the testing data that were reduced to PASC cases that had at least 28 days between their index date and the PASC diagnosis (AAUC_PheRS1_ = 0.555 [95% CI: 0.496, 0.612]). PheRS2 was only analyzed in the latter data (AAUC_PheRS2_ = 0.605 [95% CI: 0.549, 0.663]) but performed better than PheRS1, which was also evident from its pseudo-R^2^ which was almost five-fold higher (0.0116 and 0.0547, respectively). A combination score further improved the discrimination of cases and controls, but its accuracy remained low (AAUC_Combined_ = 0.615 [0.561, 0.670]; Table 2).

We also explored if PheRSs based on additional suggestively associated PheCodes (defined as *p* < 1 × 10^−3^) could further improve the prediction of PASC but found their individual or combined predictive ability slightly worse compared to the PheRSs that were based on phenome-wide significant hits (e.g., AAUC_Combined_ = 0.601 [0.548, 0.658]; Table S7).

**Figure 5:**
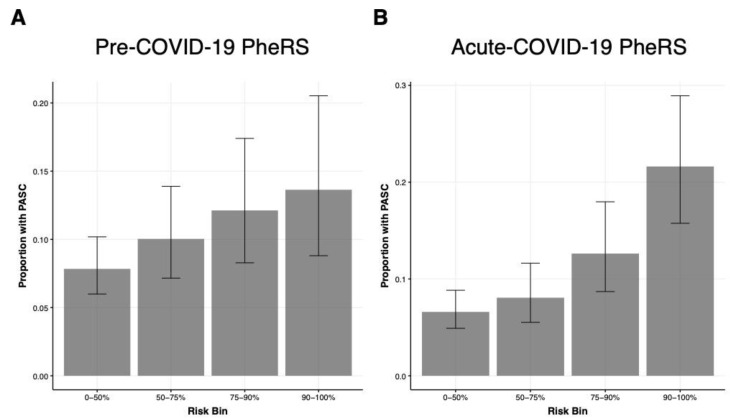
PheRS-based risk stratification in the testing data. The proportion of PASC cases among different PheRS bins is shown for (**A**) the pre-COVID-19 PheRS (PheRS1) and (**B**) the acute-COVID-19 PheRS (PheRS2). The analysis is based on patients with history of COVID-19 in 2022 with at least 28 days between the first COVID-19 and first PASC diagnosis; 123 cases and 1154 controls. Risk bins correspond to selected ranges of the PheRS distributions. Vertical lines represent confidence intervals for binomial proportions [46].

While the use for individual-level prediction seemed very limited, we found that PheRS1 and PheRS2 could significantly enrich PASC cases in their top 10% and top 10–25% risk bins compared to the lower 50% of their distributions (Table 3). For example, individuals in the top 10% of PheRS1 were 2.5 times (OR = 2.48 [95% CI: 1.24, 4.97]) and in the top 10% of PheRS2 4.1 times more likely to obtain a PASC diagnosis (OR: 4.10 [2.28, 7.40]). Moreover, both PheRSs combined improved enrichment also in the top 10–25% risk bin (OR: 2.91 [1.73, 4.90]), identifying a fourth of all COVID-19 cases with substantially increased risk for PASC (Table 3, Figure 5).

## 4. Discussion

In this study, we used data from a relatively large cohort of patients with history of COVID-19 from Michigan Medicine. We applied a PheWAS approach across time-restricted phenomes to identify phenotypes that may predispose to PASC. We found seven phenotypes (IBS, concussion, nausea and vomiting, shortness of breath, respiratory abnormalities, allergic reaction to food, and general circulatory disease) of the pre-COVID-19 period and 69 phenotypes (predominantly respiratory and circulatory symptoms) of the acute-COVID-19 period to be significantly enriched among PASC cases. Most of them were also observed enriched among PASC cases in the post-COVID19 period indicating that some of these phenotypes might have become longer-lasting or even chronic conditions. When incorporating these findings into PheRSs, we found that both the pre-COVID-19 PheRS and the acute-COVID-19 PheRS could predict PASC only with low accuracy among patients with a history of COVID-19, even when combined.

Possible explanations could be the random variation due to the small number of PASC cases, or differences due to different waves of coronavirus variants, the effect of vaccines, and changes in treatment and care of severe cases. Temporal trends in PASC diagnosis and management make this forward-looking prediction exercise much harder. We noted differences in the feature distributions between the training and testing sets, e.g., “nausea and vomiting” among the pre-COVID-19 features or “anxiety” among the acute-COVID-19 features, showed less pronounced differences between PASC cases and “No PASC” controls in the testing set (File S1J,K). However, both combined PheRSs could identify a quarter of patients with a history of COVID-19 in the testing cohort with a 3.5-fold increased risk of PASC (95% CI: 2.19, 5.55) compared to the bottom 50%. This observation highlighted the clinical utility of existing EHR data on pre-existing and acute COVID-19 symptoms for risk stratification and the identification of a large group of vulnerable individuals who might benefit from stricter protective measures or earlier interventions.

A comparison of our findings with previous studies confirmed many pre-existing conditions that are predisposed to PASC. For example, in the pre-COVID-19 period PheWAS, we identified several respiratory symptoms that predisposed to PASC, including shortness of breath and other respiratory abnormalities. These findings are consistent with previous works [15,27,50]. The literature on IBS as a pre-disposing diagnosis for PASC seems sparse; however, there might be a connection between gut microbiota and the clinical course of COVID-19 [51] and mediation of risk factors effects for COVID-19 [52,53]. Similarly, little seems to be known of concussion as a pre-disposing diagnosis for PASC; yet, pre-existing cognitive risk factors such as mild traumatic brain injury were reported as enriched among cognitive PASC cases compared to non-cognitive PASC patients [54]. Future studies are needed to substantiate our findings and investigate how pre-disposing diagnoses relate to PASC. In addition to the results from the pre-COVID-19 period conditions, our findings from the acute-COVID-19 period also accord with previous studies. Among the 69 PASC-associated phenotypes, the majority were respiratory symptoms and in line with earlier reports (e.g., cough [55,56], dyspnea [57], respiratory insufficiency [58]). Additionally, the identified muscle-related symptoms, including myalgia, malaise, and fatigue, were supported by previous PASC studies [59,60]. Similar to a previous study, we found circulatory diseases to play an essential role as a predisposing factor for PASC [61]. While not all observed associations were previously reported, our sensitivity analyses indicated overall robustness across various settings [62,63].

An overlap between the enriched symptoms in the three periods implies the possibility of PASC being recurring symptoms of pre-existing conditions [17]. The difference in subsiding rate between cases and controls in some symptoms (e.g., respiratory symptoms) potentially indicates the development of chronic conditions [9,64].

There are several limitations to our analysis. First, we focused on predisposing diagnoses and performed matching, incl. on age, gender, and race/ethnicity, to adjust for potential confounding; however, these demographic characteristics were previously implicated as pre-disposing factors [65,66,67]. So, while matching and adjusting for these covariates might have effectively increased the power to identify pre-existing phenotypes that increase the risk for PASC, we disregarded these demographic factors as PASC predictors. Future studies are needed to evaluate the combined contributions of these variables in more comprehensive prediction models. Second, although a clinical diagnosis of PASC was used, many reported symptoms are non-specific to PASC, and defining PASC consistently across the time period of this study is nearly impossible [68]. The uncertainty around the definition of PASC is reflected in an initial lack of CDC-approved ICD10 codes. For example, the code “U09.9” (“Post COVID-19 condition, unspecified”) was first introduced in October 2021, while it was recommended to also accompany this new code with existing codes for specific conditions and/or identified symptoms [69]. Before the approval of this code, the CDC encouraged providers to use an alternative but COVID-19-unrelated code, namely “B94.8” (“Sequelae of other specified infectious and parasitic diseases”) [70]. The use of PSL diagnoses enabled us to detect PASC cases before any CDC recommendations were implemented. This covers the period of March 2020 to October 2021, a pre-vaccination period where PASC incidence was possibly higher. In addition, the various descriptions in the PSL diagnoses we used to define PASC cases (see Supplementary Table S1) reflect the developing language and awareness of PASC, e.g., “Post-COVID-19 syndrome”, “COVID-19 long hauler” and “Multiple persistent symptoms after COVID-19”. Furthermore, many of the PASC-related PSL diagnoses offered specific information about the underlying conditions and symptoms.

The performed post-COVID-19 PheWAS validated our definition of PASC in that it identified many of the established PASC symptoms. Yet, the awareness about PASC only recently increased and still might lead to an underdiagnosis of PASC [71,72]. For example, we only observed 2.7% PASC-diagnosed patients in our COVID-19 positive cohort, which is far lower than PASC studies from the US, which estimated a prevalence between 19% and 35% [73]. As a result, our predictions of PASC might be overly conservative. The available diagnosis codes for PASC lacked specificity to stratify PASC cases into PASC subtypes reliably. Future studies that incorporate natural language processing of clinical notes and that have larger sample sizes will likely improve the identification of PASC cases and subtypes [74]. Third, the analysis was restricted to the patients with a history of COVID-19 who were also seen at MM during the pre-COVID-19 and post-COVID-19 periods; due to this selection bias, both cases and controls might be less healthy and older compared to randomly chosen individuals with a history of COVID-19 [75].

Moreover, it has been reported that around 15%–40% of the confirmed COVID-19 population were asymptomatic [76,77]. Using data from a health system caused our cohort to be enriched for symptomatic COVID-19 patients, while asymptomatic COVID-19 cases may be underrepresented. Such biases and omissions might limit the generalizability to the overall population. Although this study included a large size of COVID-19 patients, attention might be given to expanding and diversifying the collection and analysis of data.

Our study used a clinical definition of PASC. In addition to the commonly used ICD code U09.9 (“Post COVID-19 condition, unspecified”) or B94.8 (“Sequelae of other specified infectious and parasitic diseases”), we applied the information from the EHR internal problem list database (PSL, Table S1) to categorize PASC patients, which enabled us to collect patients whose diagnosis were recorded even before official ICD-10 recommendations/codes became available. The post-COVID-19 period PheWAS validated our PASC definition in that we enriched diagnoses consistent with subtypes of PASC that were previously reported (e.g., shortness of breath, neurological disorders, malaise, fatigue, and dysphagia) [3,74,78]. Furthermore, given the benefit of rich retrospective EHR data, we could adjust for essential confounders in our models, including race, Elixhauser comorbidity score, vaccination status, etc., that might have affected PASC outcomes. We expect that our approach and the resulting prediction models will improve over time with increasing sample sizes and, by doing so, will likely facilitate earlier detection of PASC cases or improve risk stratification. Furthermore, a better characterization of PASC mechanisms might inform on distinct PASC forms that differ in their profiles of pre-existing conditions.

## 5. Conclusions

PASC represents a worldwide public health challenge affecting millions of people. While effective therapies for PASC are still in development [79,80,81,82], prediction and risk models can help to identify individuals at increased risk for PASC and its subcategories more reliably and potentially inform preventive or therapeutic efforts.

The present research aimed to identify PASC pre-disposing diagnoses from the pre- and acute-COVID-19 medical phenomes and to explore them as predictors for PASC. We identified known and potentially novel associations across various disease categories in both phenomes. These phenotypes, when aggregated into PheRSs, have predictive properties for PASC, especially when considered for risk stratification approaches. Future studies might consider applying more complex non-linear models such as machine learning to improve prediction models. The next opportunity will be to incorporate additional, more complex data such as laboratory measurements or medication data into such prediction models, as they have proven relevant for PASC but have yet to be fully investigated [2,83,84]. The presented PheRS framework can also be adapted to explore alternative outcomes such as survival and, by doing so, offer comprehensive insights into the long-term consequences of COVID-19.

## Figures and Tables

**Table 1:** Characteristics of patients with a history of COVID-19, stratified into patients with a PASC diagnosis (cases) and without observed PASC diagnosis (controls). Case–control matching was based on nearest neighbor matching (age at index date, pre-test years in EHR, post-test years in EHR) and exact matching (gender, primary care at MM, race/ethnicity, quarter of year at COVID-19 index date).

	COVID-19 Patients with PASC Diagnosis	COVID-19 Patients without PASC Diagnosis			
		Unmatched	*p* Value *	Matched	*p* Value *
*n*	1724	61951		17205	
Age at index date; mean (SD)	47.88 (18.85)	41.67 (22.14)	<0.001	47.12 (18.94)	0.110
Pre-test years in EHR; mean (SD)	11.70 (7.47)	10.41 (7.49)	<0.001	11.67 (7.37)	0.870
Post-test years in EHR; mean (SD)	1.07 (0.56)	0.93 (0.55)	<0.001	1.05 (0.55)	0.445
Female; *n* (%)	1112 (64.5)	35713 (57.6)	<0.001	11089 (64.5)	0.989
Primary care at MM; *n* (%)	1047 (60.7)	28773 (46.4)	<0.001	10435 (60.7)	0.969
Race/ethnicity; *n* (%)			0.151		0.990
Caucasian/Non-Hispanic	1273 (73.8)	44822 (72.4)		12730 (74.0)	
African American/Non-Hispanic	199 (11.5)	7020 (11.3)		1990 (11.6)	
Other/Non-Hispanic or Hispanic	175 (10.2)	6593 (10.6)		1746 (10.1)	
Other/unknown ethnicity	77 (4.5)	3516 (5.7)		739 (4.3)	
Quarter of year at index date; *n* (%)			<0.001		1.000
2020/1	27 (1.6)	588 (0.9)		263 (1.5)	
2020/2	57 (3.3)	1697 (2.7)		555 (3.2)	
2020/3	64 (3.7)	2617 (4.2)		640 (3.7)	
2020/4	273 (15.8)	13317 (21.5)		2730 (15.9)	
2021/1	236 (13.7)	7063 (11.4)		2360 (13.7)	
2021/2	241 (14.0)	5475 (8.8)		2410 (14.0)	
2021/3	168 (9.7)	4088 (6.6)		1680 (9.8)	
2021/4	282 (16.4)	10853 (17.5)		2820 (16.4)	
2022/1	268 (15.5)	10887 (17.6)		2680 (15.6)	
2022/2	100 (5.8)	5008 (8.1)		1000 (5.8)	
2022/3	8 (0.5)	358 (0.6)		67 (0.4)	
Neighborhood Deprivation Index (%)			0.003		0.350
Quartile 1	631 (36.6)	22679 (36.6)		6629 (38.5)	
Quartile 2	401 (23.3)	13028 (21.0)		3708 (21.6)	
Quartile 3	325 (18.9)	11330 (18.3)		3203 (18.6)	
Quartile 4	253 (14.7)	9235 (14.9)		2444 (14.2)	
Missing	114 (6.6)	5679 (9.2)		1221 (7.1)	
Population density (%)			0.002		0.128
Quartile 1	413 (24.0)	15218 (24.6)		4417 (25.7)	
Quartile 2	491 (28.5)	17796 (28.7)		5013 (29.1)	
Quartile 3	551 (32.0)	18123 (29.3)		5229 (30.4)	
Quartile 4	155 (9.0)	5135 (8.3)		1325 (7.7)	
Missing	114 (6.6)	5679 (9.2)		1221 (7.1)	
Elixhauser Score AHRQ; mean (SD)	4.52 (12.97)	3.75 (10.72)	0.003	4.01 (11.36)	0.077

* *p*-value of differences between COVID-19 patients with a PASC diagnosis and COVID-19 patients without a PASC diagnosis. Abbreviations: EHR, electronic health records; MM, Michigan Medicine; AHRQ, Agency for Healthcare Research and Quality

**Table 2:** PheRS Evaluation in the testing data (COVID-19 positive in 2022). PheRS1 was based on the significant hits of the PheWAS with the pre-COVID-19 training data (1256 cases and 11,674 controls; COVID-19 positive in 2020/2021) while PheRS2 was based on the significant hits of the PheWAS with the acute-COVID-19 training data (874 cases and 8144 controls; COVID-19 positive in 2020/2021 and at least 28 days between first COVID-19 and first PASC diagnosis). Underlying weights can be found in File S1J and Table S8.

Predictor	Testing Data		AAUC ^a^ (95% CI)	Pseudo-R^2 b^	Brier Score
	*n* Cases	*n* Controls			
PheRS1	349	3248	0.548 (0.516, 0.580)	n/a ^c^	n/a ^c^
PheRS1	123	1154	0.555 (0.496, 0.612)	0.0116	0.0857
PheRS2			0.605 (0.549, 0.663)	0.0547	0.0823
PheRS1 and PheRS2			0.615 (0.561, 0.670)	0.0553	0.0824

^a^ Adjusted for age at index date, gender, race/ethnicity, Elixhauser Score, population density, NDI, health care worker status, vaccination status, pre-test years in EHR, and severity; ^b^ Nagelkerke (Cragg and Uhler)); ^c^ not applicable, only useful in evaluating multiple models predicting the same outcome on the same dataset.

**Table 3:** PheRS-based risk stratification in the testing data. Analysis is based on patients with a history of COVID-19 in 2022 with at least 28 days between the first COVID-19 and the first PASC diagnosis; 123 cases and 1154 controls.

PheRS	Upper Risk Bin	%Cases in Risk Bin	%Cases in Lower 50%	OR (95% CI) ^a^	*p*
PheRS1	25–50%	10.0	7.8	1.48 (0.91, 2.42)	0.12
	10–25%	12.1		1.86 (1.06, 3.25)	0.029
	≥10%	13.6		2.48 (1.24, 4.97)	0.011
	≥25%	12.7		2.10 (1.29, 3.43)	0.0029
PheRS2	25–50%	8.1	6.6	1.26 (0.76, 2.08)	0.38
	10–25%	12.6		2.13 (1.25, 3.62)	0.0053
	≥10%	21.6		4.10 (2.28, 7.40)	2.7 × 10^−6^
	≥25%	16.5		2.92 (1.85, 4.59)	3.9 × 10^−6^
PheRS1 and PheRS2	25–50%	8.3	6.2	1.36 (0.82, 2.28)	0.23
	10–25%	15.2		2.91 (1.73, 4.90)	5.8 × 10^−5^
	≥10%	19.4		3.94 (2.10, 7.42)	2.1 × 10^−5^
	≥25%	17.0		3.48 (2.19, 5.55)	1.5 × 10^−7^

^a^ Enrichment of PASC cases in risk bin compared to lower 50%; adjusted for age at index date, gender, race/ethnicity, Elixhauser Score, population density, NDI, health care worker status, vaccination status, pre-test years in EHR, and severity.

## Data Availability

Data cannot be shared publicly due to patient confidentiality. The data underlying the results presented in the study are available from the University of Michigan Precision Health Analytics Platform at https://precisionhealth.umich.edu/tools-resources/data-access-tools/ (last accessed: 3 February 2023) for researchers who meet the criteria for access to confidential data.

## Supplementary Materials

The following supporting information can be downloaded at: https://www.mdpi.com/article/10.3390/jcm12041328/s1, Figure S1. The proportion of clinically documented PASC within 3 months of testing positive and the number of total unmatched COVID-19-positive individuals by year quarter when they were tested positive/diagnosed for COVID-19 for the first time; Figure S2. Overview flowchart showing the sample filtering and analysis setup; Figure S3. Forest plots of the PreCOVID-19 Sensitivity analyses; Figure S4. Forest plots of the Acute COVID-19 Sensitivity analyses; Figure S5. Comparison of PheCode prevalence during pre-COVID-19, acute/short-COVID-19, and post-COVID-19 periods in cases and controls; Table S1. PASC Problem list; Table S2. PASC symptom and concurrent symptom mapping; Table S3. Covariate summary and missingness in the unmatched and matched cohort; Table S4. Main and sensitivity PheWAS; Table S5. Concurrent diagnoses on day of the first PASC diagnosis; Table S6. Enrichment 29 known PASC symptoms among post-COVID-19 diagnoses in PASC cases compared to “No PASC” controls; Table S7. PheRS Evaluation in the testing data (COVID-19 positive in 2022); Table S8. Weights for combining PheRS1 and PheRS2 or PheRS1* and PheRS2*; File S1A. Post-COVID-19 (6 months) PheWAS; File S1B. Pre-COVID-19 PheWAS; File S1C. Acute-COVID-19 PheWAS; File S1D. Sensitivity Analysis “Severity” Pre-COVID-19; File S1E. Sensitivity Analysis “Gender” Pre-COVID-19; File S1F. Sensitivity Analysis “Year of infection” Pre-COVID-19; File S1G. Sensitivity Analysis “Severity” Acute-COVID-19; File S1H. Sensitivity Analysis “Gender” Acute-COVID-19; File S1I. Sensitivity Analysis “Year of Infection” Acute-COVID-19; File S1J. PheRS Weights.

## Appendix A

### Appendix A.1. Neighborhood Disadvantage Index (NDI)

The neighborhood disadvantage index (NDI) without the proportion of Black includes four census indicators (proportion of female-headed families with children, the proportion of households with public assistance income or food stamps; the proportion of families with income below the federal poverty level; the proportion of population age 16+ unemployed). We did not include measures of racial distribution within this index.

### Appendix A.2. Pre- and Post-COVID-19 Years in EHR

For each individual, we defined pre-COVID-19 years in EHR as the time between the first recorded EHR entry and the first positive COVID-19 test or diagnosis and post-COVID-19 years in EHR as the time between the first positive COVID-19 test or diagnosis and the last recorded EHR entry.

### Appendix A.3. Vaccination Status

We created a covariate to capture the vaccination status at the index date coded as “unvaccinated”, “after 1. vaccination”, “after full vaccination” and “after booster” using records of vaccinations for patients who received a vaccination at MM or who have a recorded vaccination record in the Michigan Care Improvement Registry (MCIR). Michigan’s immunization providers are required to report COVID vaccination to MCIR within 24 h of administration, meaning the EHR vaccination record should be nearly complete. Among the matched case–control cohort, 11,925 individuals had at the date of their first positive test or COVID-19 diagnosis no documented vaccination and thus were considered unvaccinated. It is possible although unlikely that they may have been vaccinated elsewhere and these records were not available. A total of 7004 individuals had at least one documented dose of a COVID-19 vaccine. According to FDA’s vaccination guideline [85], we categorized 6000 individuals as fully vaccinated in the primary series, meaning documentation of two doses of Moderna or Pfizer-BioNTech vaccine, or a single dose of Janssen vaccine at least 21 days before the corresponding test date [86,87,88]. A subset of 1646 of the fully vaccinated patients was further classified as being boosted, i.e., they received at least 1 additional vaccination at least 21 days after completing the primary series. The remaining 1004 vaccinated patients who did not complete the primary series were considered “partially vaccinated”.

### Appendix A.4. COVID-19 Severity

The covariate for COVID-19-related outcome severity was dichotomized as “severe”, i.e., either hospitalization or intensive care unit (ICU) admission within one month after a positive SARS-CoV-2 RT-PCR test result or COVID-19 diagnosis, or death within two months after a positive RT-PCR test or COVID-19 diagnosis. Data on hospitalizations, ICU admissions, and death were obtained from Michigan Medicine’s EHR databases as well as the Michigan Death Registry. The remaining individuals were considered “non-severe” COVID-19-related outcomes and included non-hospitalized, symptomatic, or asymptomatic COVID-19 cases.

### Appendix A.5. Elixhauser comorbidity score

The Elixhauser comorbidity score developed by the Agency for Healthcare Research and Quality (AHRQ) was calculated to comprehensively characterize patients’ pre-existing comorbidity conditions using ICD9 and ICD10 codes and the R package “comorbidity” [37,38].

### Appendix A.6. Healthcare Worker (HCW) Status

Healthcare worker (HCW) status was defined based on documented participation in an HCW survey or a SARS-CoV-2 PCR test order for HCW.
