# Supplementary material for: Characterizing and Predicting Post-Acute Sequelae of SARS CoV-2 Infection (PASC) in a Large Academic Medical Center in the US
Source: J Clin Med. 2023 Feb 7;12(4):1328. doi: 10.3390/jcm12041328 (PMC9967320; doi:10.3390/jcm12041328)
Supplement: Supplementary file 1 [file jcm-12-01328-s001.zip › jcm-2083245-supplementary.pdf]

Journal of
Clinical Medicine

MDPI

SUPPLEMENTARY MATERIALS

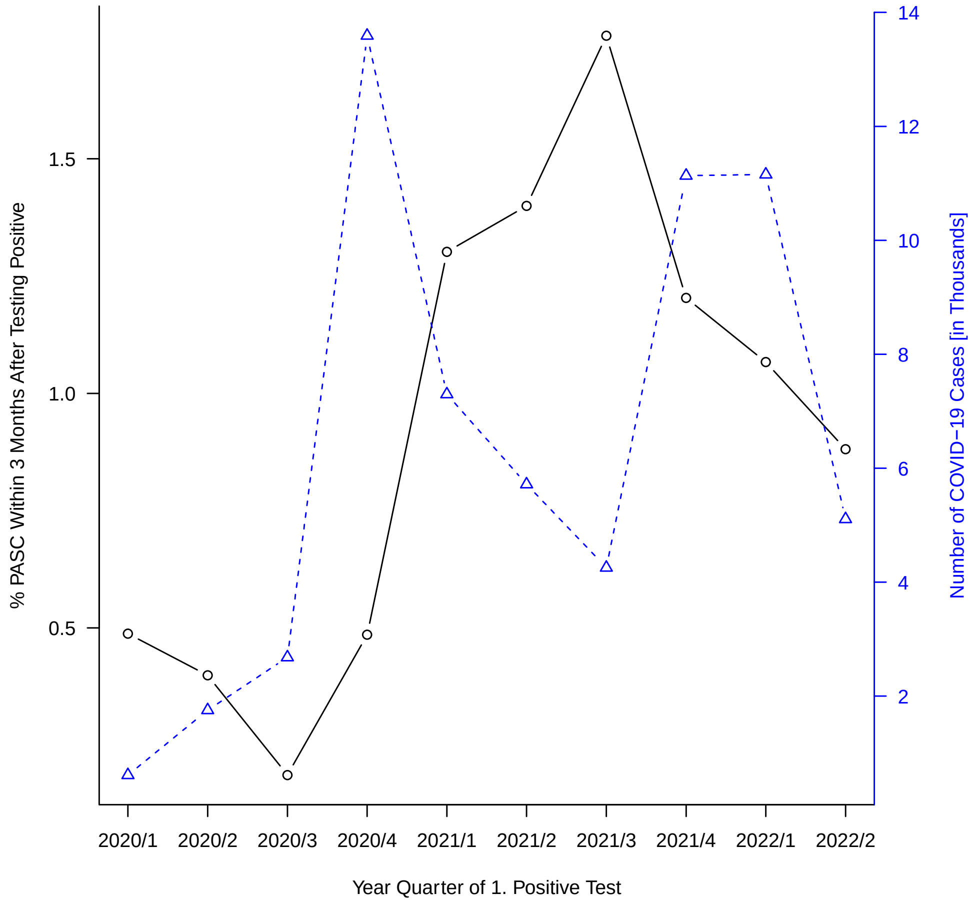

Figure S1. The proportion of clinically documented PASC within 3 months of testing positive and the number of total unmatched COVID-19-positive individuals by year quarter when they were tested positive/diagnosed for COVID-19 for the first time.

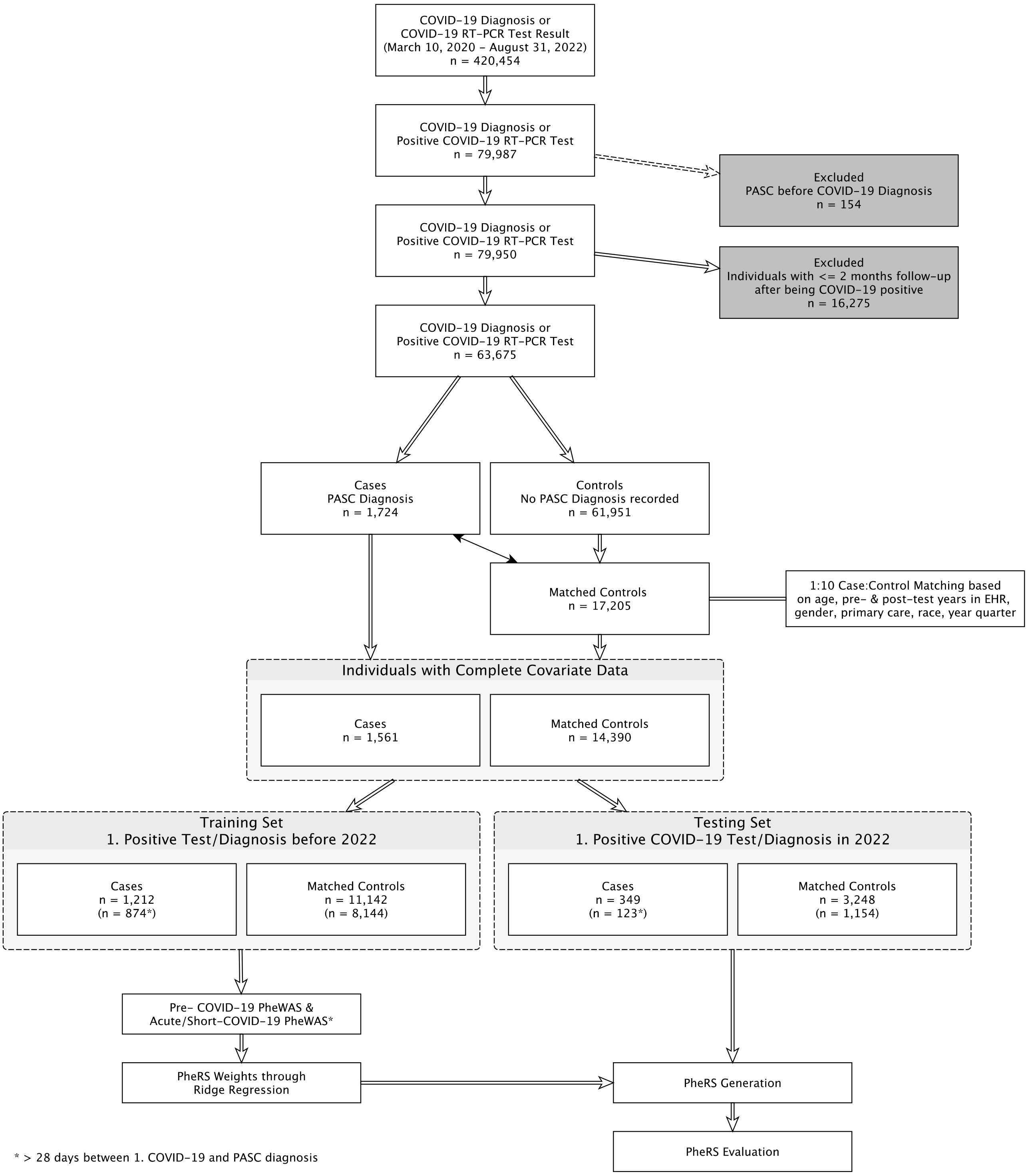

Figure S2. Overview flowchart showing the sample filtering and analysis setup.

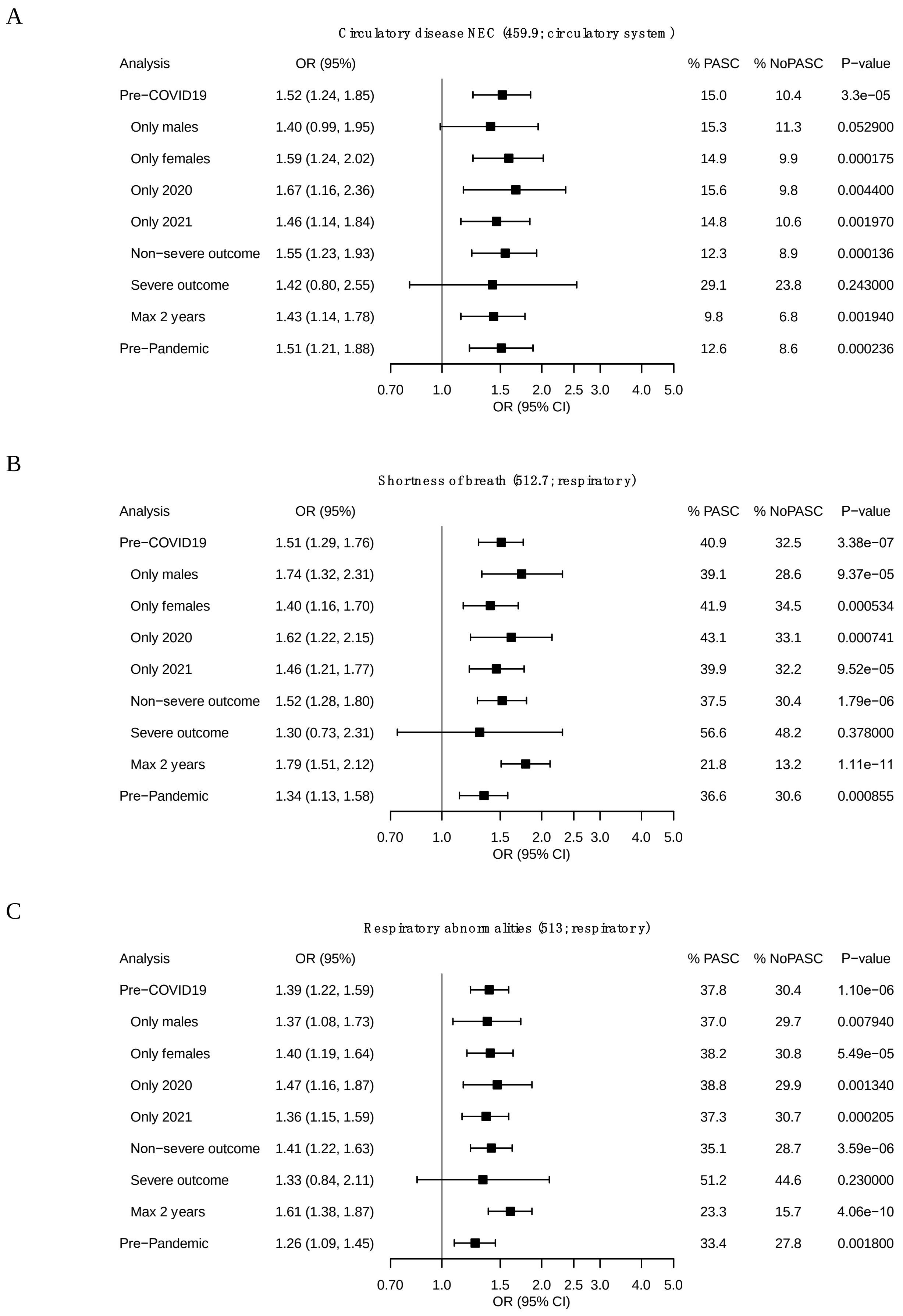
A
Circulatory disease NEC (459.9; circulatory system)
Analysis OR (95%) % PASC % NoPASC P−value
Pre−COVID19 1.52 (1.24, 1.85) 15.0 10.4 3.3e−05
Only males 1.40 (0.99, 1.95) 15.3 11.3 0.052900
Only females 1.59 (1.24, 2.02) 14.9 9.9 0.000175
Only 2020 1.67 (1.16, 2.36) 15.6 9.8 0.004400
Only 2021 1.46 (1.14, 1.84) 14.8 10.6 0.001970
Non−severe outcome 1.55 (1.23, 1.93) 12.3 8.9 0.000136
Severe outcome 1.42 (0.80, 2.55) 29.1 23.8 0.243000
Max 2 years 1.43 (1.14, 1.78) 9.8 6.8 0.001940
Pre−Pandemic 1.51 (1.21, 1.88) 12.6 8.6 0.000236
0.70 1.0 1.5 2.0 2.5 3.0 4.0 5.0
OR (95% CI)
B
Shortness of breath (512.7; respiratory)
Analysis OR (95%) % PASC % NoPASC P−value
Pre−COVID19 1.51 (1.29, 1.76) 40.9 32.5 3.38e−07
Only males 1.74 (1.32, 2.31) 39.1 28.6 9.37e−05
Only females 1.40 (1.16, 1.70) 41.9 34.5 0.000534
Only 2020 1.62 (1.22, 2.15) 43.1 33.1 0.000741
Only 2021 1.46 (1.21, 1.77) 39.9 32.2 9.52e−05
Non−severe outcome 1.52 (1.28, 1.80) 37.5 30.4 1.79e−06
Severe outcome 1.30 (0.73, 2.31) 56.6 48.2 0.378000
Max 2 years 1.79 (1.51, 2.12) 21.8 13.2 1.11e−11
Pre−Pandemic 1.34 (1.13, 1.58) 36.6 30.6 0.000855
0.70 1.0 1.5 2.0 2.5 3.0 4.0 5.0
OR (95% CI)
C
Respiratory abnormalities (513; respiratory)
Analysis OR (95%) % PASC % NoPASC P−value
Pre−COVID19 1.39 (1.22, 1.59) 37.8 30.4 1.10e−06
Only males 1.37 (1.08, 1.73) 37.0 29.7 0.007940
Only females 1.40 (1.19, 1.64) 38.2 30.8 5.49e−05
Only 2020 1.47 (1.16, 1.87) 38.8 29.9 0.001340
Only 2021 1.36 (1.15, 1.59) 37.3 30.7 0.000205
Non−severe outcome 1.41 (1.22, 1.63) 35.1 28.7 3.59e−06
Severe outcome 1.33 (0.84, 2.11) 51.2 44.6 0.230000
Max 2 years 1.61 (1.38, 1.87) 23.3 15.7 4.06e−10
Pre−Pandemic 1.26 (1.09, 1.45) 33.4 27.8 0.001800
0.70 1.0 1.5 2.0 2.5 3.0 4.0 5.0
OR (95% CI)

D

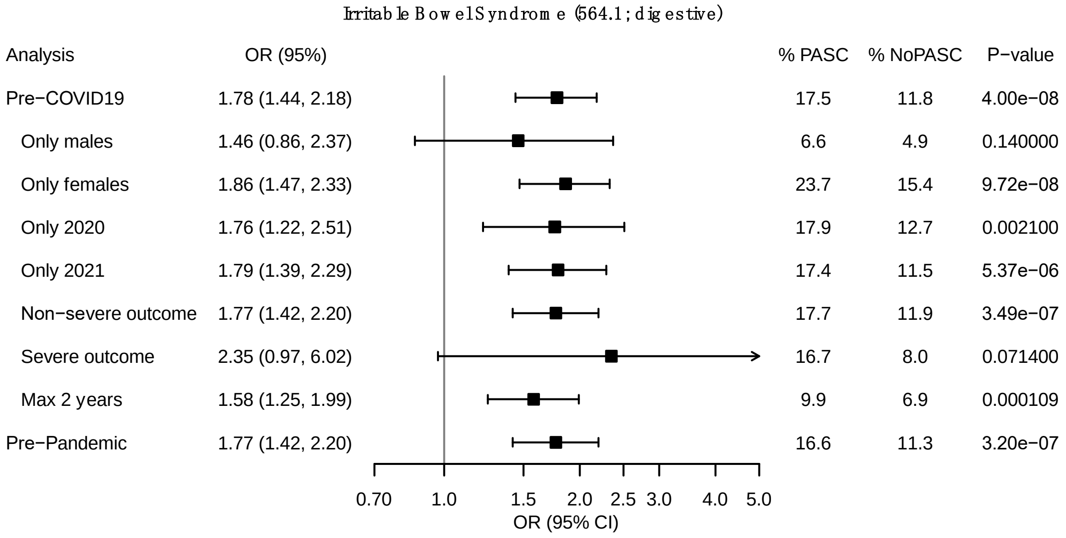
Irritable Bowel Syndrome (564.1; digestive)
Analysis OR (95%) % PASC % NoPASC P−value
Pre−COVID19 1.78 (1.44, 2.18) 17.5 11.8 4.00e−08
Only males 1.46 (0.86, 2.37) 6.6 4.9 0.140000
Only females 1.86 (1.47, 2.33) 23.7 15.4 9.72e−08
Only 2020 1.76 (1.22, 2.51) 17.9 12.7 0.002100
Only 2021 1.79 (1.39, 2.29) 17.4 11.5 5.37e−06
Non−severe outcome 1.77 (1.42, 2.20) 17.7 11.9 3.49e−07
Severe outcome 2.35 (0.97, 6.02) 16.7 8.0 0.071400
Max 2 years 1.58 (1.25, 1.99) 9.9 6.9 0.000109
Pre−Pandemic 1.77 (1.42, 2.20) 16.6 11.3 3.20e−07
0.70 1.0 1.5 2.0 2.5 3.0 4.0 5.0
OR (95% CI)

E

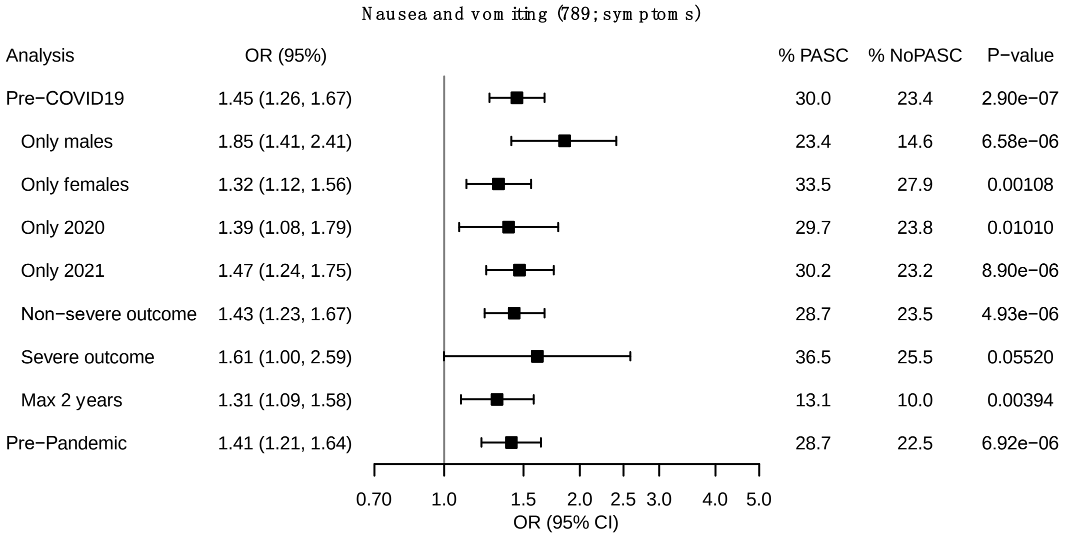
Nausea and vomiting (789; symptoms)
Analysis OR (95%) % PASC % NoPASC P−value
Pre−COVID19 1.45 (1.26, 1.67) 30.0 23.4 2.90e−07
Only males 1.85 (1.41, 2.41) 23.4 14.6 6.58e−06
Only females 1.32 (1.12, 1.56) 33.5 27.9 0.00108
Only 2020 1.39 (1.08, 1.79) 29.7 23.8 0.01010
Only 2021 1.47 (1.24, 1.75) 30.2 23.2 8.90e−06
Non−severe outcome 1.43 (1.23, 1.67) 28.7 23.5 4.93e−06
Severe outcome 1.61 (1.00, 2.59) 36.5 25.5 0.05520
Max 2 years 1.31 (1.09, 1.58) 13.1 10.0 0.00394
Pre−Pandemic 1.41 (1.21, 1.64) 28.7 22.5 6.92e−06
0.70 1.0 1.5 2.0 2.5 3.0 4.0 5.0
OR (95% CI)

F

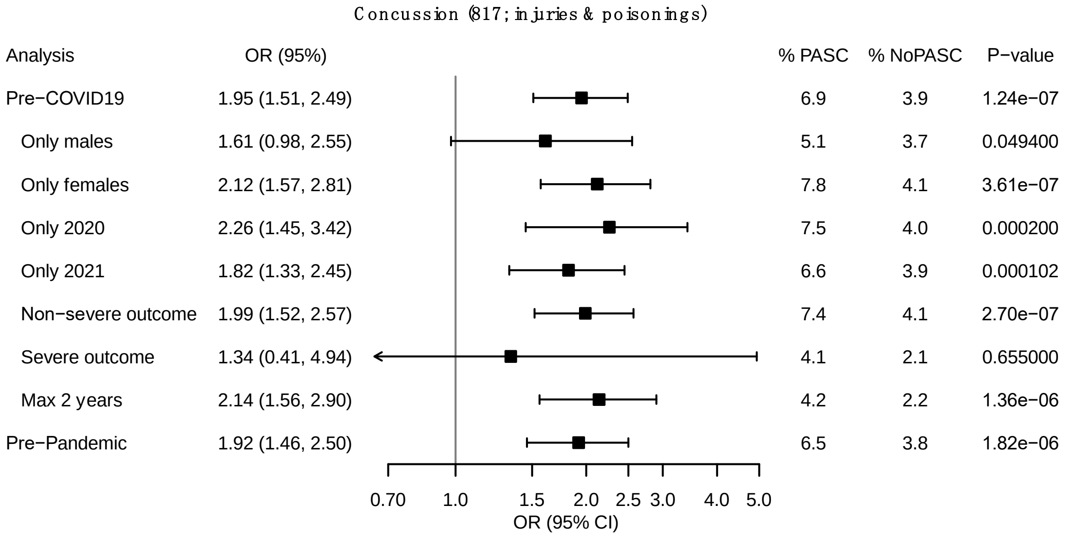
Concussion (817; injuries & poisonings)
Analysis OR (95%) % PASC % NoPASC P−value
Pre−COVID19 1.95 (1.51, 2.49) 6.9 3.9 1.24e−07
Only males 1.61 (0.98, 2.55) 5.1 3.7 0.049400
Only females 2.12 (1.57, 2.81) 7.8 4.1 3.61e−07
Only 2020 2.26 (1.45, 3.42) 7.5 4.0 0.000200
Only 2021 1.82 (1.33, 2.45) 6.6 3.9 0.000102
Non−severe outcome 1.99 (1.52, 2.57) 7.4 4.1 2.70e−07
Severe outcome 1.34 (0.41, 4.94) 4.1 2.1 0.655000
Max 2 years 2.14 (1.56, 2.90) 4.2 2.2 1.36e−06
Pre−Pandemic 1.92 (1.46, 2.50) 6.5 3.8 1.82e−06
0.70 1.0 1.5 2.0 2.5 3.0 4.0 5.0
OR (95% CI)

G

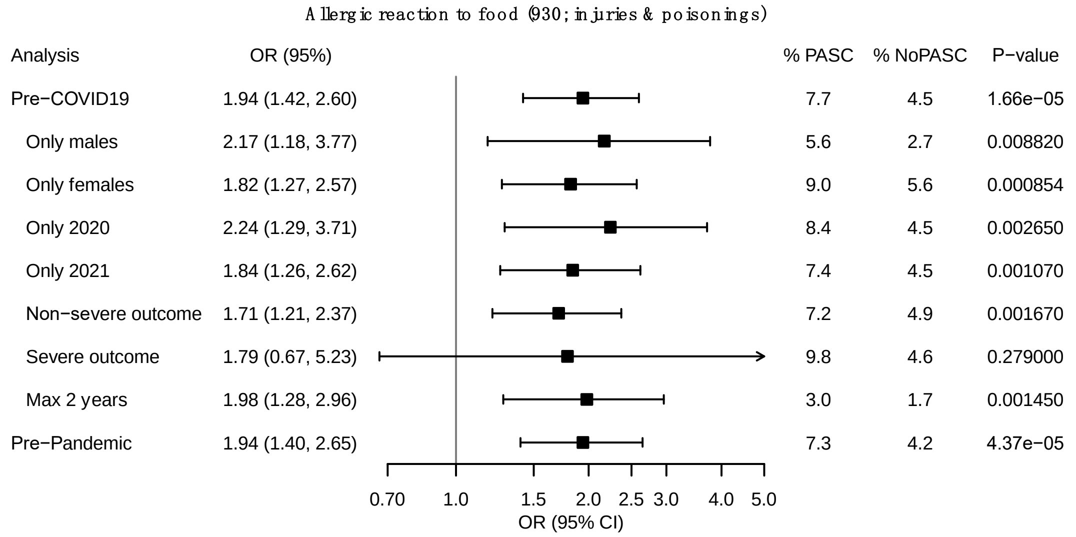

Figure S3. A-G. Forest plots of the PreCOVID-19 Sensitivity analyses. Effect sizes and PheCode frequencies in cases and controls of significantly associated PheCodes are shown. Pre-COVID-19: main analysis, only males, only females, COVID-19 positive in 2020, COVID-19 positive in 2021, non-severe COVID-19 outcome, severe COVID-19 outcome, max 2 years before COVID-19 diagnosis, and before 2020. Of significantly associated parent/child PheCodes only the phecode with the stronger association signal is shown. Sample sizes of each analysis can be found in **Table S5**.

A

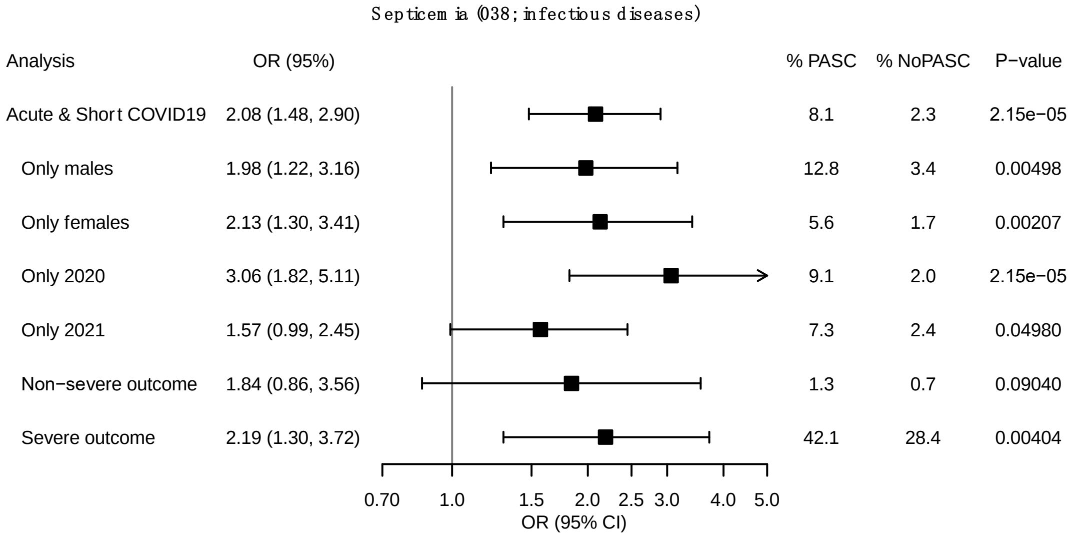

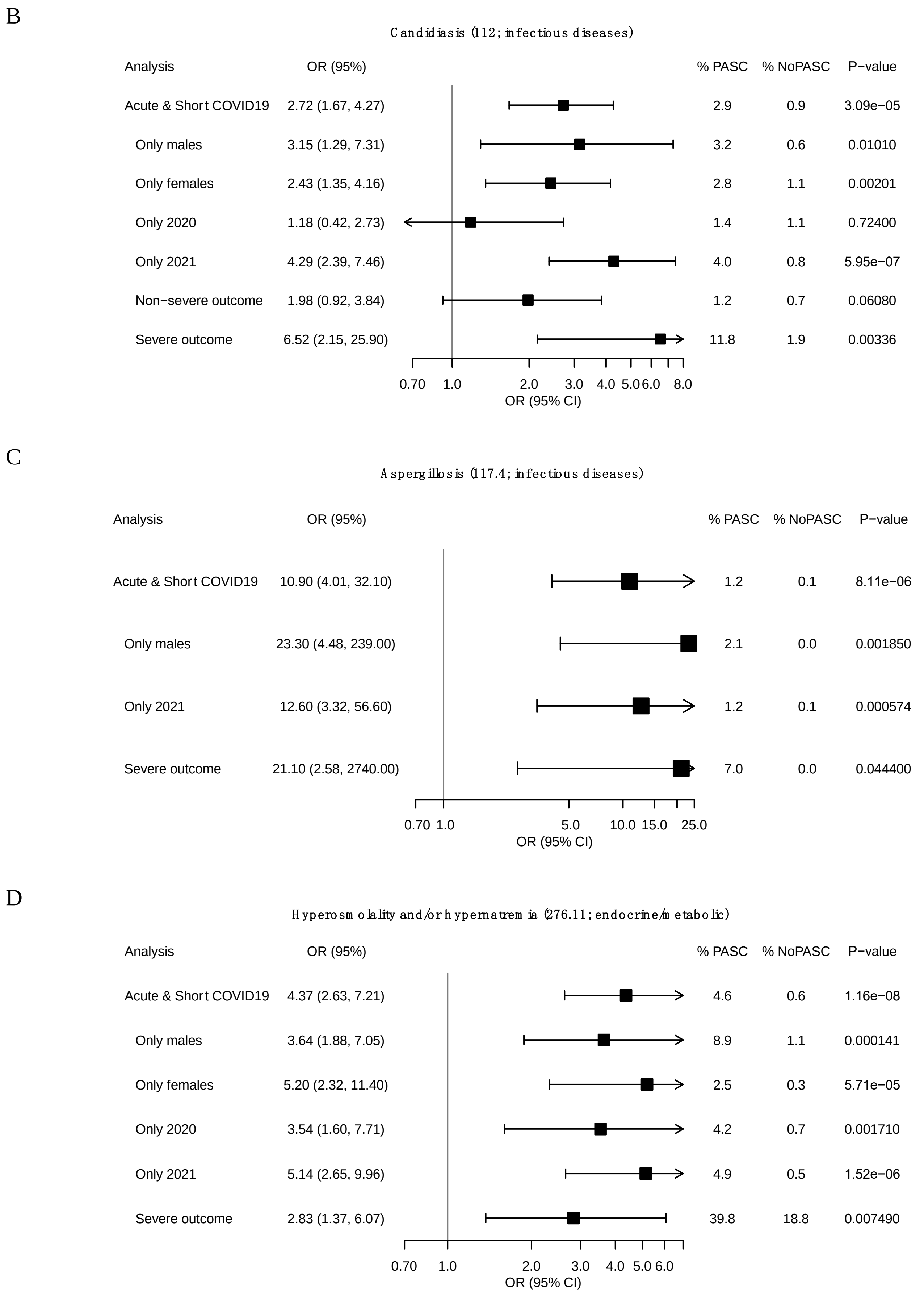
B
Candidiasis (112; infectious diseases)
Analysis
OR (95%)
% PASC
% NoPASC
P−value
Acute & Short COVID19 2.72 (1.67, 4.27) 2.9 0.9 3.09e−05
Only males 3.15 (1.29, 7.31) 3.2 0.6 0.01010
Only females 2.43 (1.35, 4.16) 2.8 1.1 0.00201
Only 2020 1.18 (0.42, 2.73) 1.4 1.1 0.72400
Only 2021 4.29 (2.39, 7.46) 4.0 0.8 5.95e−07
Non−severe outcome 1.98 (0.92, 3.84) 1.2 0.7 0.06080
Severe outcome 6.52 (2.15, 25.90) 11.8 1.9 0.00336
0.70 1.0 2.0 3.0 4.0 5.0 6.0 8.0
OR (95% CI)
C
Aspergillosis (117.4; infectious diseases)
Analysis
OR (95%)
% PASC
% NoPASC
P−value
Acute & Short COVID19 10.90 (4.01, 32.10) 1.2 0.1 8.11e−06
Only males 23.30 (4.48, 239.00) 2.1 0.0 0.001850
Only 2021 12.60 (3.32, 56.60) 1.2 0.1 0.000574
Severe outcome 21.10 (2.58, 2740.00) 7.0 0.0 0.044400
0.70 1.0 5.0 10.0 15.0 25.0
OR (95% CI)
D
Hyperosmolality and/or hypernatremia (276.11; endocrine/metabolic)
Analysis
OR (95%)
% PASC
% NoPASC
P−value
Acute & Short COVID19 4.37 (2.63, 7.21) 4.6 0.6 1.16e−08
Only males 3.64 (1.88, 7.05) 8.9 1.1 0.000141
Only females 5.20 (2.32, 11.40) 2.5 0.3 5.71e−05
Only 2020 3.54 (1.60, 7.71) 4.2 0.7 0.001710
Only 2021 5.14 (2.65, 9.96) 4.9 0.5 1.52e−06
Severe outcome 2.83 (1.37, 6.07) 39.8 18.8 0.007490
0.70 1.0 2.0 3.0 4.0 5.0 6.0
OR (95% CI)

E

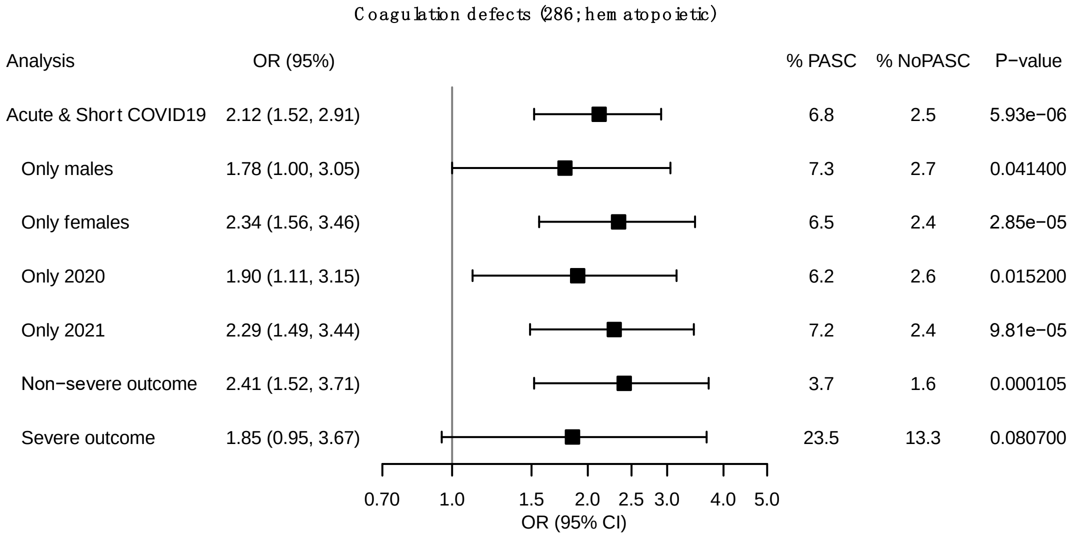
Coagulation defects (286; hematopoietic)
Analysis
OR (95%)
% PASC
% NoPASC
P−value
Acute & Short COVID19 2.12 (1.52, 2.91) 6.8 2.5 5.93e−06
Only males 1.78 (1.00, 3.05) 7.3 2.7 0.041400
Only females 2.34 (1.56, 3.46) 6.5 2.4 2.85e−05
Only 2020 1.90 (1.11, 3.15) 6.2 2.6 0.015200
Only 2021 2.29 (1.49, 3.44) 7.2 2.4 9.81e−05
Non−severe outcome 2.41 (1.52, 3.71) 3.7 1.6 0.000105
Severe outcome 1.85 (0.95, 3.67) 23.5 13.3 0.080700
0.70 1.0 1.5 2.0 2.5 3.0 4.0 5.0
OR (95% CI)

F

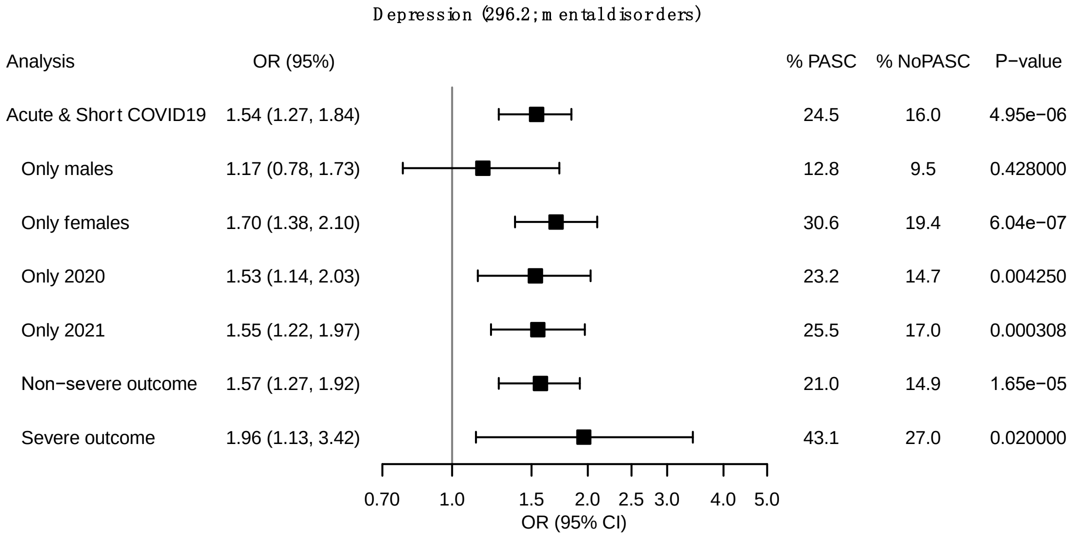
Depression (296.2; mental disorders)
Analysis
OR (95%)
% PASC
% NoPASC
P−value
Acute & Short COVID19 1.54 (1.27, 1.84) 24.5 16.0 4.95e−06
Only males 1.17 (0.78, 1.73) 12.8 9.5 0.428000
Only females 1.70 (1.38, 2.10) 30.6 19.4 6.04e−07
Only 2020 1.53 (1.14, 2.03) 23.2 14.7 0.004250
Only 2021 1.55 (1.22, 1.97) 25.5 17.0 0.000308
Non−severe outcome 1.57 (1.27, 1.92) 21.0 14.9 1.65e−05
Severe outcome 1.96 (1.13, 3.42) 43.1 27.0 0.020000
0.70 1.0 1.5 2.0 2.5 3.0 4.0 5.0
OR (95% CI)

G

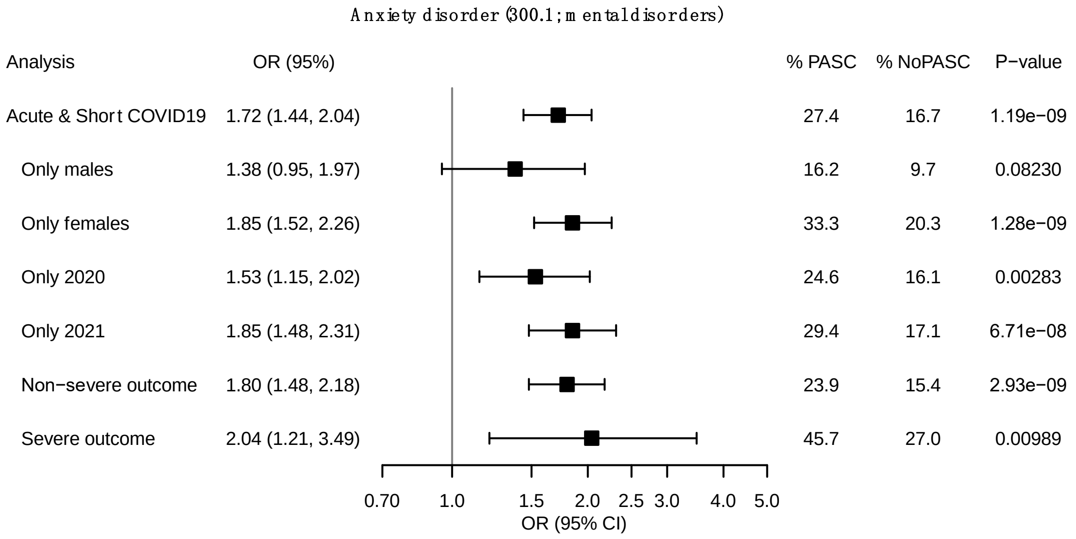
Anxiety disorder (300.1; mental disorders)
Analysis
OR (95%)
% PASC
% NoPASC
P−value
Acute & Short COVID19 1.72 (1.44, 2.04) 27.4 16.7 1.19e−09
Only males 1.38 (0.95, 1.97) 16.2 9.7 0.08230
Only females 1.85 (1.52, 2.26) 33.3 20.3 1.28e−09
Only 2020 1.53 (1.15, 2.02) 24.6 16.1 0.00283
Only 2021 1.85 (1.48, 2.31) 29.4 17.1 6.71e−08
Non−severe outcome 1.80 (1.48, 2.18) 23.9 15.4 2.93e−09
Severe outcome 2.04 (1.21, 3.49) 45.7 27.0 0.00989
0.70 1.0 1.5 2.0 2.5 3.0 4.0 5.0
OR (95% CI)

H

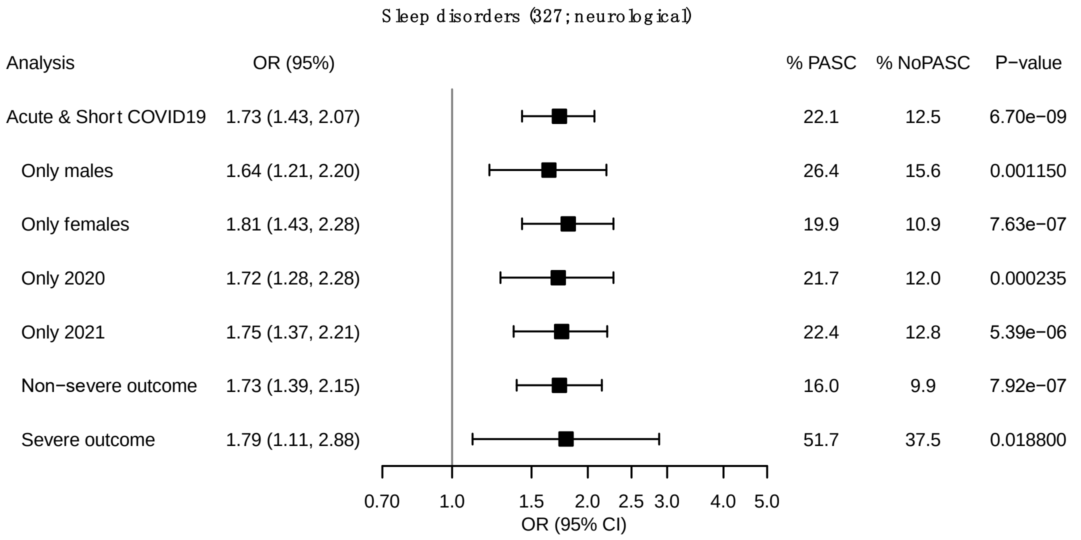
Sleep disorders (327; neurological)
Analysis
OR (95%)
% PASC
% NoPASC
P−value
Acute & Short COVID19
1.73 (1.43, 2.07)
22.1
12.5
6.70e−09
Only males
1.64 (1.21, 2.20)
26.4
15.6
0.001150
Only females
1.81 (1.43, 2.28)
19.9
10.9
7.63e−07
Only 2020
1.72 (1.28, 2.28)
21.7
12.0
0.000235
Only 2021
1.75 (1.37, 2.21)
22.4
12.8
5.39e−06
Non−severe outcome
1.73 (1.39, 2.15)
16.0
9.9
7.92e−07
Severe outcome
1.79 (1.11, 2.88)
51.7
37.5
0.018800
0.70
1.0
1.5
2.0
2.5
3.0
4.0
5.0
OR (95% CI)

I

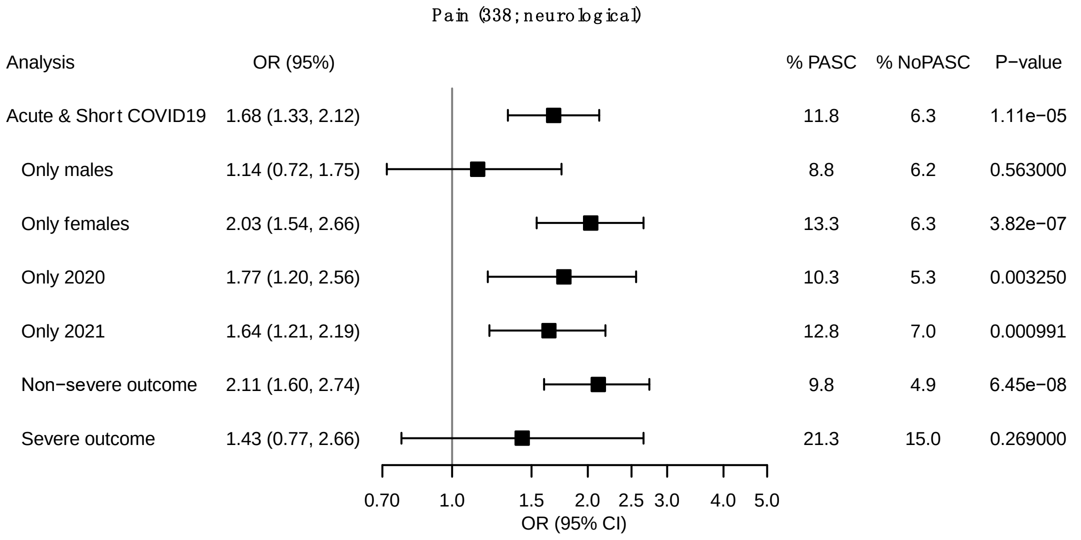
Pain (338; neurological)
Analysis
OR (95%)
% PASC
% NoPASC
P−value
Acute & Short COVID19
1.68 (1.33, 2.12)
11.8
6.3
1.11e−05
Only males
1.14 (0.72, 1.75)
8.8
6.2
0.563000
Only females
2.03 (1.54, 2.66)
13.3
6.3
3.82e−07
Only 2020
1.77 (1.20, 2.56)
10.3
5.3
0.003250
Only 2021
1.64 (1.21, 2.19)
12.8
7.0
0.000991
Non−severe outcome
2.11 (1.60, 2.74)
9.8
4.9
6.45e−08
Severe outcome
1.43 (0.77, 2.66)
21.3
15.0
0.269000
0.70
1.0
1.5
2.0
2.5
3.0
4.0
5.0
OR (95% CI)

J

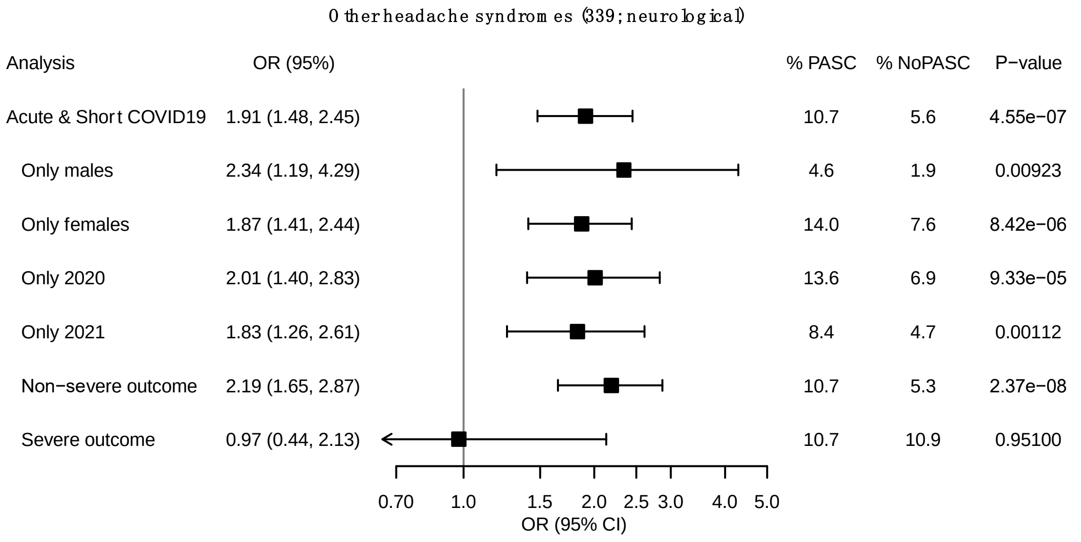
Other headache syndromes (339; neurological)
Analysis
OR (95%)
% PASC
% NoPASC
P−value
Acute & Short COVID19
1.91 (1.48, 2.45)
10.7
5.6
4.55e−07
Only males
2.34 (1.19, 4.29)
4.6
1.9
0.00923
Only females
1.87 (1.41, 2.44)
14.0
7.6
8.42e−06
Only 2020
2.01 (1.40, 2.83)
13.6
6.9
9.33e−05
Only 2021
1.83 (1.26, 2.61)
8.4
4.7
0.00112
Non−severe outcome
2.19 (1.65, 2.87)
10.7
5.3
2.37e−08
Severe outcome
0.97 (0.44, 2.13)
10.7
10.9
0.95100
0.70
1.0
1.5
2.0
2.5
3.0
4.0
5.0
OR (95% CI)

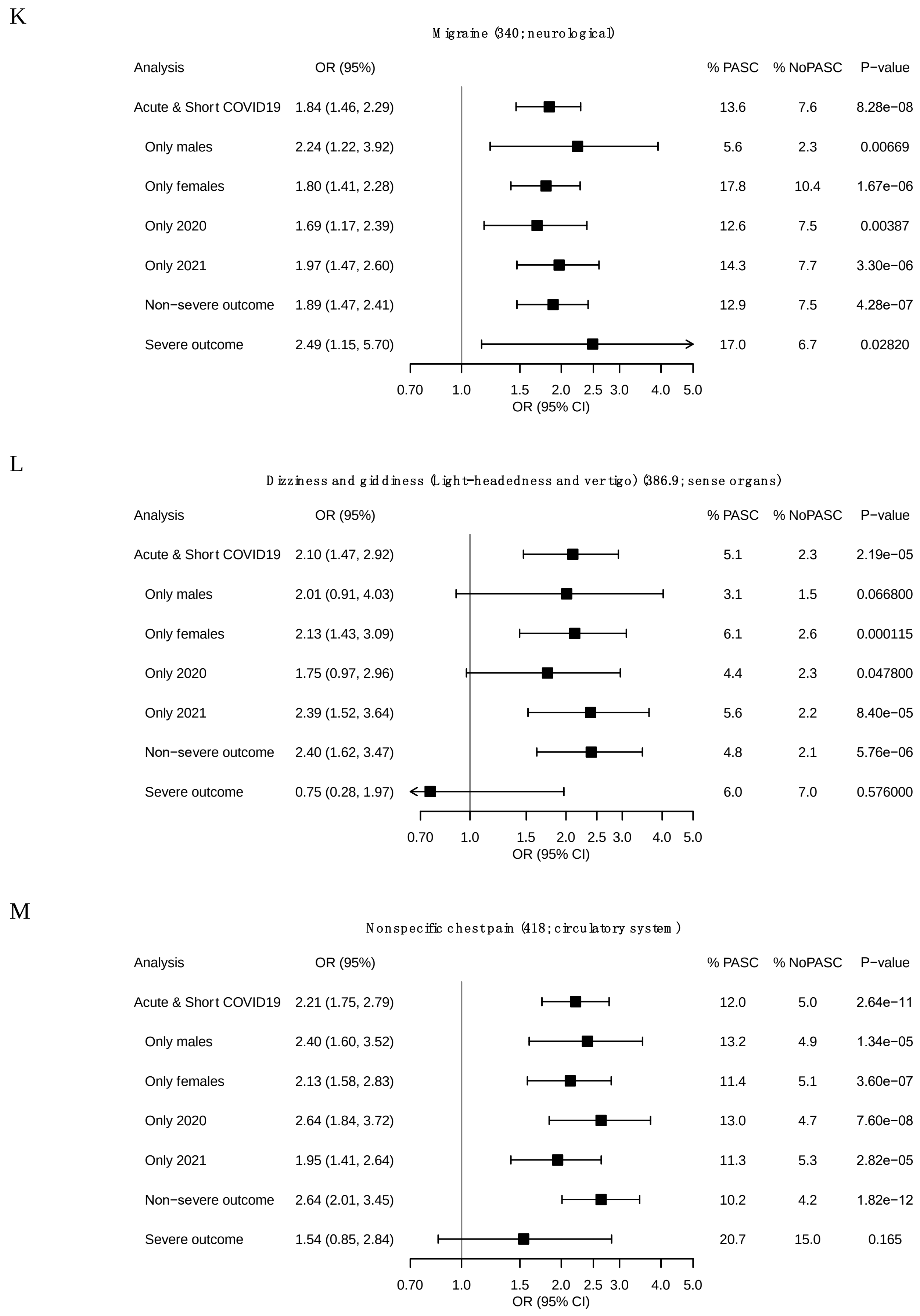
K
Migraine (340; neurological)
Analysis OR (95%) % PASC % NoPASC P−value
Acute & Short COVID19 1.84 (1.46, 2.29) 13.6 7.6 8.28e−08
Only males 2.24 (1.22, 3.92) 5.6 2.3 0.00669
Only females 1.80 (1.41, 2.28) 17.8 10.4 1.67e−06
Only 2020 1.69 (1.17, 2.39) 12.6 7.5 0.00387
Only 2021 1.97 (1.47, 2.60) 14.3 7.7 3.30e−06
Non−severe outcome 1.89 (1.47, 2.41) 12.9 7.5 4.28e−07
Severe outcome 2.49 (1.15, 5.70) 17.0 6.7 0.02820
0.70 1.0 1.5 2.0 2.5 3.0 4.0 5.0
OR (95% CI)
L
Dizziness and giddiness (Light-headedness and vertigo) (386.9; sense organs)
Analysis OR (95%) % PASC % NoPASC P−value
Acute & Short COVID19 2.10 (1.47, 2.92) 5.1 2.3 2.19e−05
Only males 2.01 (0.91, 4.03) 3.1 1.5 0.066800
Only females 2.13 (1.43, 3.09) 6.1 2.6 0.000115
Only 2020 1.75 (0.97, 2.96) 4.4 2.3 0.047800
Only 2021 2.39 (1.52, 3.64) 5.6 2.2 8.40e−05
Non−severe outcome 2.40 (1.62, 3.47) 4.8 2.1 5.76e−06
Severe outcome 0.75 (0.28, 1.97) 6.0 7.0 0.576000
0.70 1.0 1.5 2.0 2.5 3.0 4.0 5.0
OR (95% CI)
M
Nonspecific chest pain (418; circulatory system)
Analysis OR (95%) % PASC % NoPASC P−value
Acute & Short COVID19 2.21 (1.75, 2.79) 12.0 5.0 2.64e−11
Only males 2.40 (1.60, 3.52) 13.2 4.9 1.34e−05
Only females 2.13 (1.58, 2.83) 11.4 5.1 3.60e−07
Only 2020 2.64 (1.84, 3.72) 13.0 4.7 7.60e−08
Only 2021 1.95 (1.41, 2.64) 11.3 5.3 2.82e−05
Non−severe outcome 2.64 (2.01, 3.45) 10.2 4.2 1.82e−12
Severe outcome 1.54 (0.85, 2.84) 20.7 15.0 0.165
0.70 1.0 1.5 2.0 2.5 3.0 4.0 5.0
OR (95% CI)

N

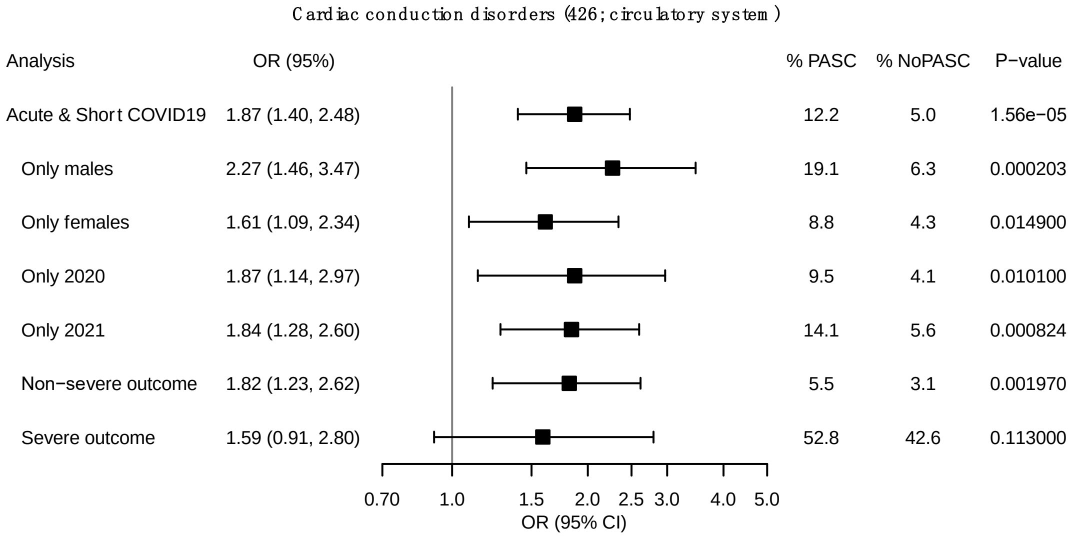
Cardiac conduction disorders (426; circulatory system)
Analysis
OR (95%)
% PASC
% NoPASC
P−value
Acute & Short COVID19 1.87 (1.40, 2.48) 12.2 5.0 1.56e−05
Only males 2.27 (1.46, 3.47) 19.1 6.3 0.000203
Only females 1.61 (1.09, 2.34) 8.8 4.3 0.014900
Only 2020 1.87 (1.14, 2.97) 9.5 4.1 0.010100
Only 2021 1.84 (1.28, 2.60) 14.1 5.6 0.000824
Non−severe outcome 1.82 (1.23, 2.62) 5.5 3.1 0.001970
Severe outcome 1.59 (0.91, 2.80) 52.8 42.6 0.113000
0.70 1.0 1.5 2.0 2.5 3.0 4.0 5.0
OR (95% CI)

O

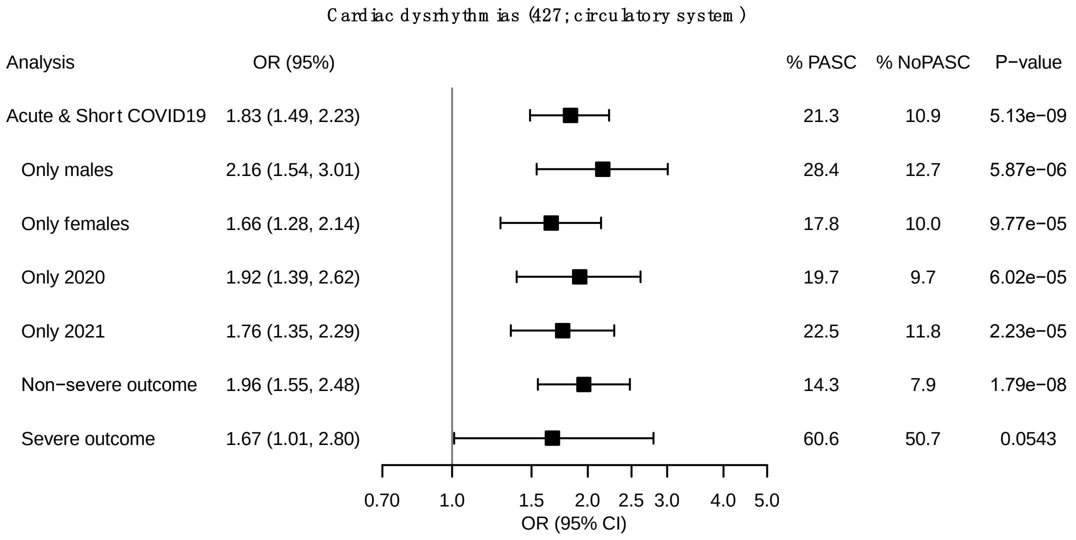
Cardiac dysrhythmias (427; circulatory system)
Analysis
OR (95%)
% PASC
% NoPASC
P−value
Acute & Short COVID19 1.83 (1.49, 2.23) 21.3 10.9 5.13e−09
Only males 2.16 (1.54, 3.01) 28.4 12.7 5.87e−06
Only females 1.66 (1.28, 2.14) 17.8 10.0 9.77e−05
Only 2020 1.92 (1.39, 2.62) 19.7 9.7 6.02e−05
Only 2021 1.76 (1.35, 2.29) 22.5 11.8 2.23e−05
Non−severe outcome 1.96 (1.55, 2.48) 14.3 7.9 1.79e−08
Severe outcome 1.67 (1.01, 2.80) 60.6 50.7 0.0543
0.70 1.0 1.5 2.0 2.5 3.0 4.0 5.0
OR (95% CI)

P

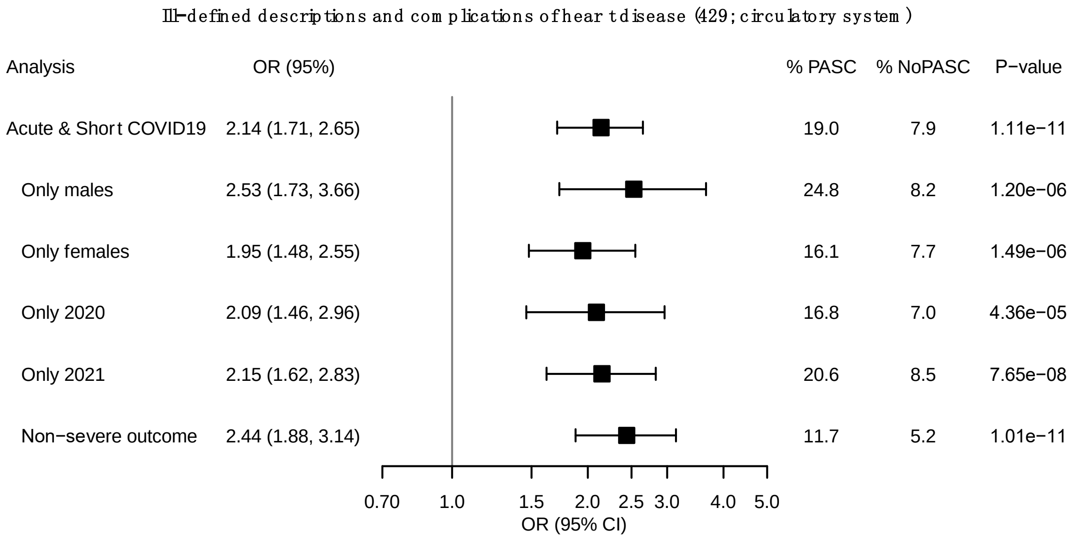
Ill−defined descriptions and complications of heart disease (429; circulatory system)
Analysis
OR (95%)
% PASC
% NoPASC
P−value
Acute & Short COVID19 2.14 (1.71, 2.65) 19.0 7.9 1.11e−11
Only males 2.53 (1.73, 3.66) 24.8 8.2 1.20e−06
Only females 1.95 (1.48, 2.55) 16.1 7.7 1.49e−06
Only 2020 2.09 (1.46, 2.96) 16.8 7.0 4.36e−05
Only 2021 2.15 (1.62, 2.83) 20.6 8.5 7.65e−08
Non−severe outcome 2.44 (1.88, 3.14) 11.7 5.2 1.01e−11
0.70 1.0 1.5 2.0 2.5 3.0 4.0 5.0
OR (95% CI)

Q

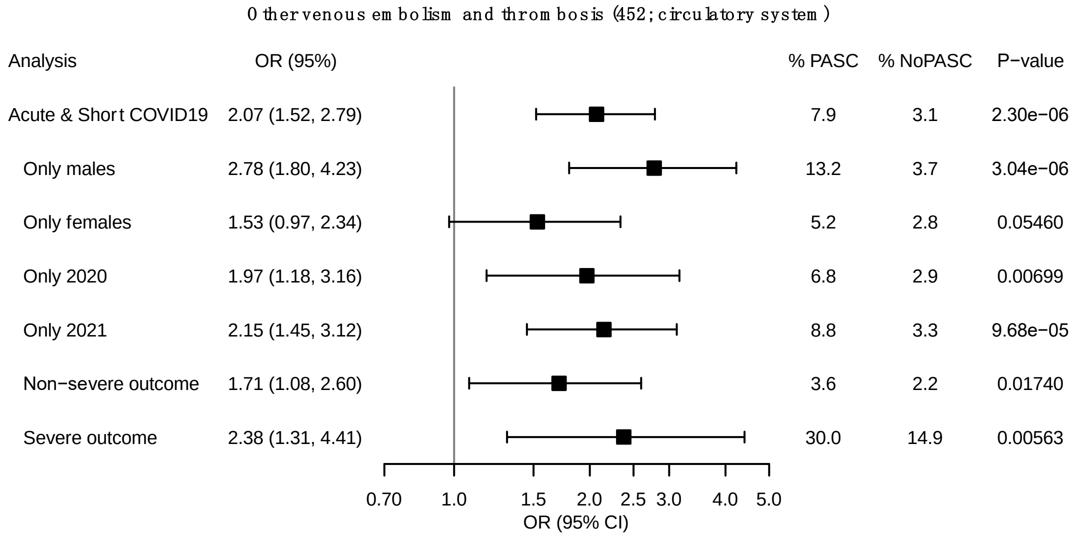
Other venous embolism and thrombosis (452; circulatory system)
Analysis
OR (95%)
% PASC
% NoPASC
P−value
Acute & Short COVID19 2.07 (1.52, 2.79) 7.9 3.1 2.30e−06
Only males 2.78 (1.80, 4.23) 13.2 3.7 3.04e−06
Only females 1.53 (0.97, 2.34) 5.2 2.8 0.05460
Only 2020 1.97 (1.18, 3.16) 6.8 2.9 0.00699
Only 2021 2.15 (1.45, 3.12) 8.8 3.3 9.68e−05
Non−severe outcome 1.71 (1.08, 2.60) 3.6 2.2 0.01740
Severe outcome 2.38 (1.31, 4.41) 30.0 14.9 0.00563
0.70 1.0 1.5 2.0 2.5 3.0 4.0 5.0
OR (95% CI)

R

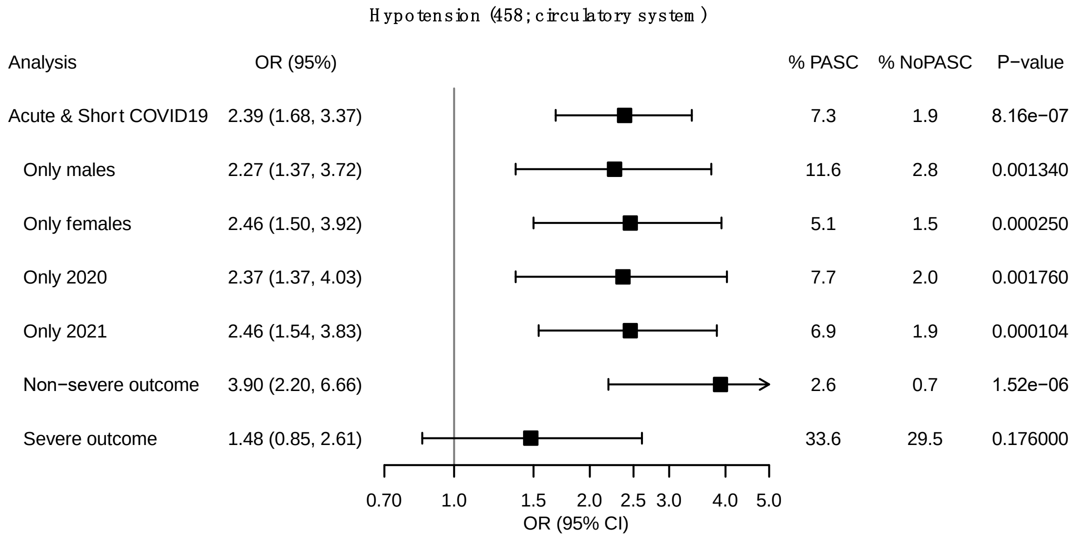
Hypotension (458; circulatory system)
Analysis
OR (95%)
% PASC
% NoPASC
P−value
Acute & Short COVID19 2.39 (1.68, 3.37) 7.3 1.9 8.16e−07
Only males 2.27 (1.37, 3.72) 11.6 2.8 0.001340
Only females 2.46 (1.50, 3.92) 5.1 1.5 0.000250
Only 2020 2.37 (1.37, 4.03) 7.7 2.0 0.001760
Only 2021 2.46 (1.54, 3.83) 6.9 1.9 0.000104
Non−severe outcome 3.90 (2.20, 6.66) 2.6 0.7 1.52e−06
Severe outcome 1.48 (0.85, 2.61) 33.6 29.5 0.176000
0.70 1.0 1.5 2.0 2.5 3.0 4.0 5.0
OR (95% CI)

S

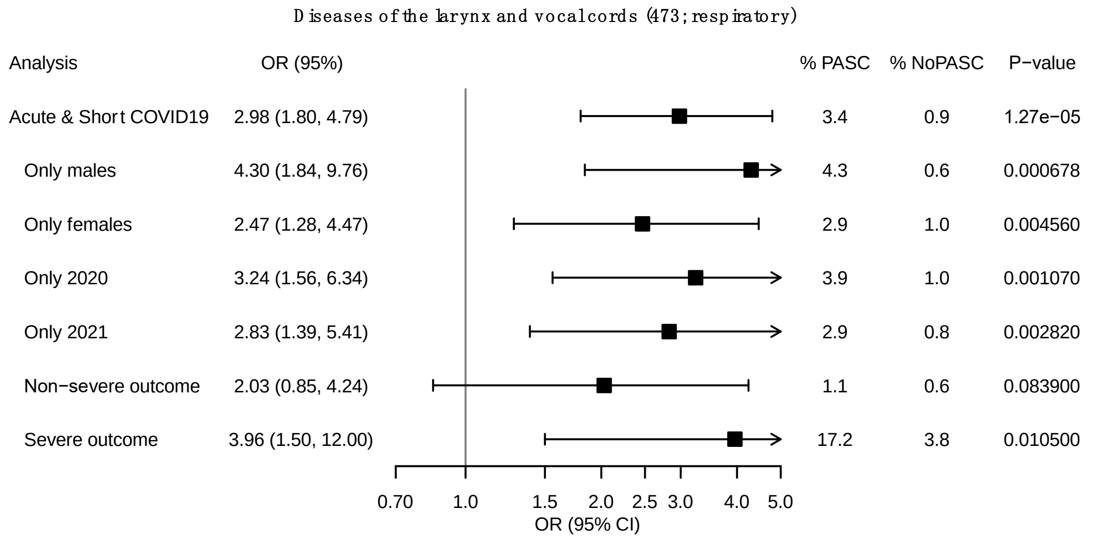
Diseases of the larynx and vocal cords (473; respiratory)
Analysis
OR (95%)
% PASC
% NoPASC
P−value
Acute & Short COVID19 2.98 (1.80, 4.79) 3.4 0.9 1.27e−05
Only males 4.30 (1.84, 9.76) 4.3 0.6 0.000678
Only females 2.47 (1.28, 4.47) 2.9 1.0 0.004560
Only 2020 3.24 (1.56, 6.34) 3.9 1.0 0.001070
Only 2021 2.83 (1.39, 5.41) 2.9 0.8 0.002820
Non−severe outcome 2.03 (0.85, 4.24) 1.1 0.6 0.083900
Severe outcome 3.96 (1.50, 12.00) 17.2 3.8 0.010500
0.70 1.0 1.5 2.0 2.5 3.0 4.0 5.0
OR (95% CI)

T

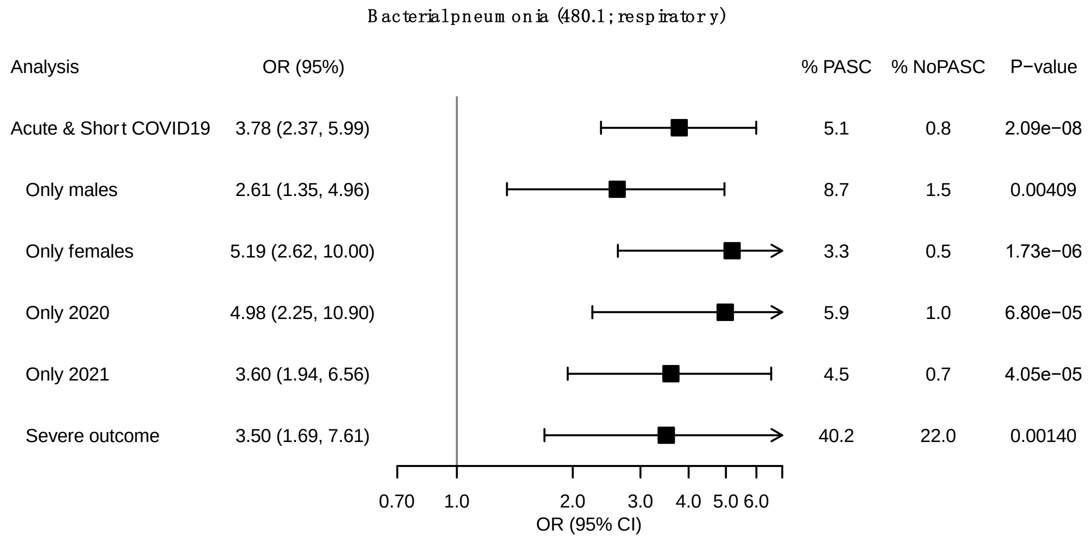
Bacterial pneumonia (480.1; respiratory)
Analysis
OR (95%)
% PASC
% NoPASC
P−value
Acute & Short COVID19
3.78 (2.37, 5.99)
5.1
0.8
2.09e−08
Only males
2.61 (1.35, 4.96)
8.7
1.5
0.00409
Only females
5.19 (2.62, 10.00)
3.3
0.5
1.73e−06
Only 2020
4.98 (2.25, 10.90)
5.9
1.0
6.80e−05
Only 2021
3.60 (1.94, 6.56)
4.5
0.7
4.05e−05
Severe outcome
3.50 (1.69, 7.61)
40.2
22.0
0.00140
0.70
1.0
2.0
3.0
4.0
5.0
6.0
OR (95% CI)

U

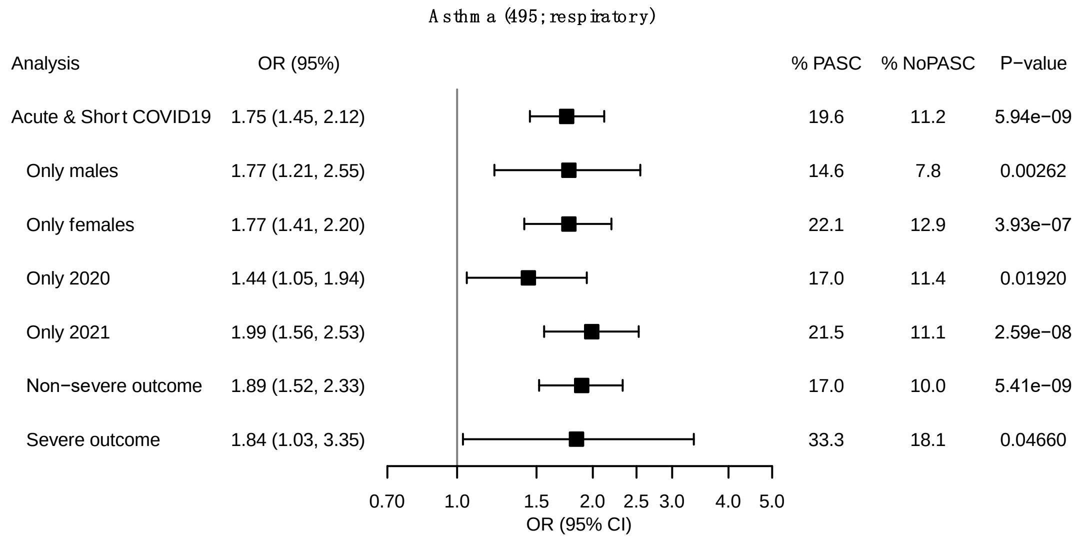
Asthma (495; respiratory)
Analysis
OR (95%)
% PASC
% NoPASC
P−value
Acute & Short COVID19
1.75 (1.45, 2.12)
19.6
11.2
5.94e−09
Only males
1.77 (1.21, 2.55)
14.6
7.8
0.00262
Only females
1.77 (1.41, 2.20)
22.1
12.9
3.93e−07
Only 2020
1.44 (1.05, 1.94)
17.0
11.4
0.01920
Only 2021
1.99 (1.56, 2.53)
21.5
11.1
2.59e−08
Non−severe outcome
1.89 (1.52, 2.33)
17.0
10.0
5.41e−09
Severe outcome
1.84 (1.03, 3.35)
33.3
18.1
0.04660
0.70
1.0
1.5
2.0
2.5
3.0
4.0
5.0
OR (95% CI)

V

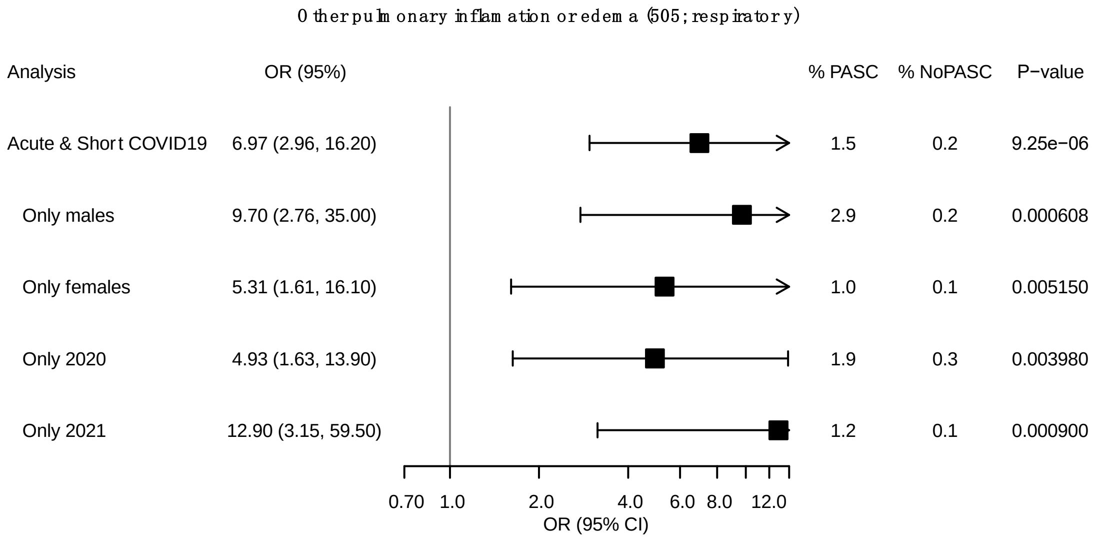
Other pulmonary inflammation or edema (505; respiratory)
Analysis
OR (95%)
% PASC
% NoPASC
P−value
Acute & Short COVID19
6.97 (2.96, 16.20)
1.5
0.2
9.25e−06
Only males
9.70 (2.76, 35.00)
2.9
0.2
0.000608
Only females
5.31 (1.61, 16.10)
1.0
0.1
0.005150
Only 2020
4.93 (1.63, 13.90)
1.9
0.3
0.003980
Only 2021
12.90 (3.15, 59.50)
1.2
0.1
0.000900
0.70
1.0
2.0
4.0
6.0
8.0
12.0
OR (95% CI)

W

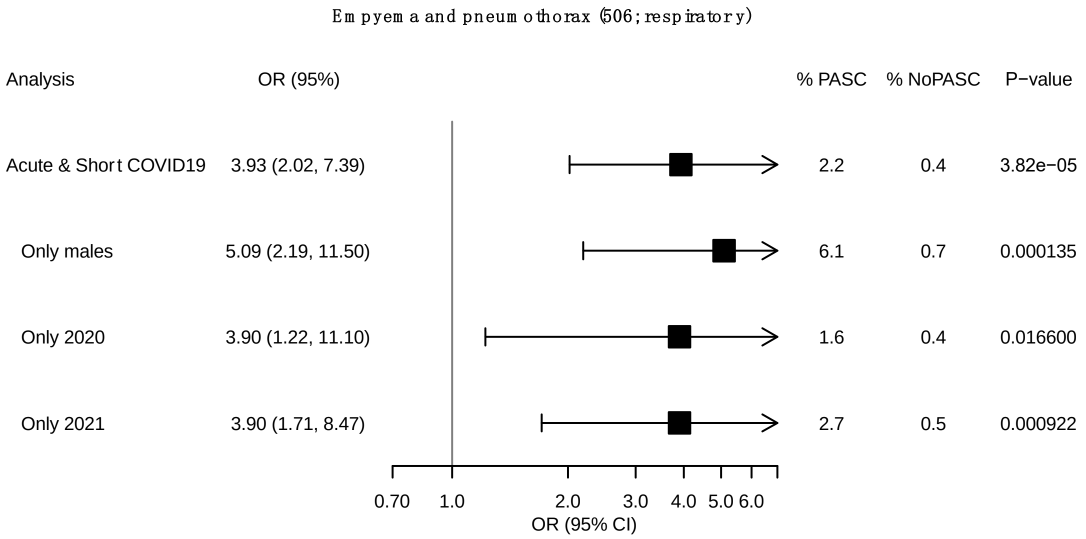
Empyema and pneumothorax (506; respiratory)
Analysis
OR (95%)
% PASC
% NoPASC
P−value
Acute & Short COVID19 3.93 (2.02, 7.39) 2.2 0.4 3.82e−05
Only males 5.09 (2.19, 11.50) 6.1 0.7 0.000135
Only 2020 3.90 (1.22, 11.10) 1.6 0.4 0.016600
Only 2021 3.90 (1.71, 8.47) 2.7 0.5 0.000922
0.70 1.0 2.0 3.0 4.0 5.0 6.0
OR (95% CI)

X

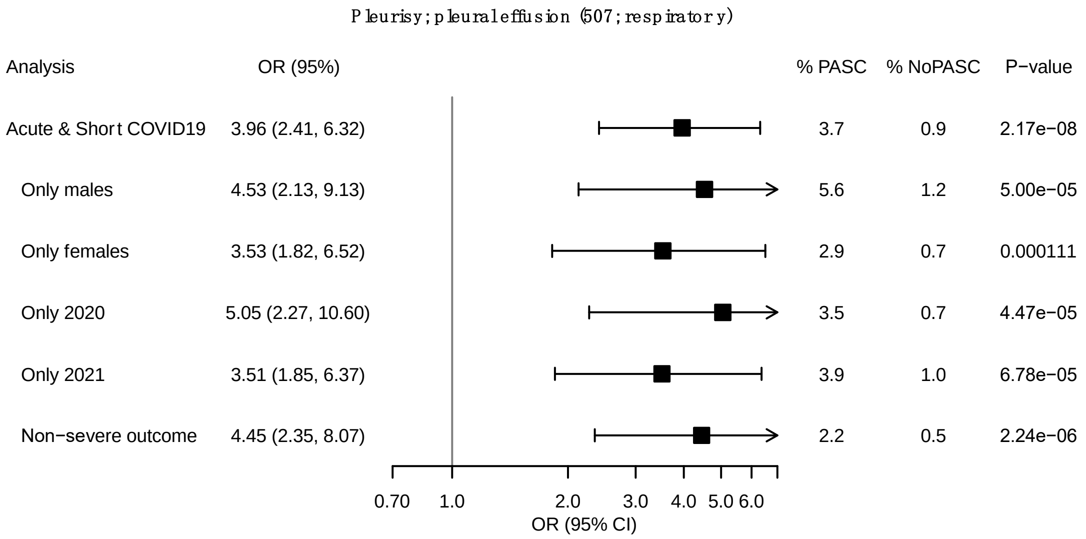
Pleurisy; pleural effusion (507; respiratory)
Analysis
OR (95%)
% PASC
% NoPASC
P−value
Acute & Short COVID19 3.96 (2.41, 6.32) 3.7 0.9 2.17e−08
Only males 4.53 (2.13, 9.13) 5.6 1.2 5.00e−05
Only females 3.53 (1.82, 6.52) 2.9 0.7 0.000111
Only 2020 5.05 (2.27, 10.60) 3.5 0.7 4.47e−05
Only 2021 3.51 (1.85, 6.37) 3.9 1.0 6.78e−05
Non−severe outcome 4.45 (2.35, 8.07) 2.2 0.5 2.24e−06
0.70 1.0 2.0 3.0 4.0 5.0 6.0
OR (95% CI)

Y

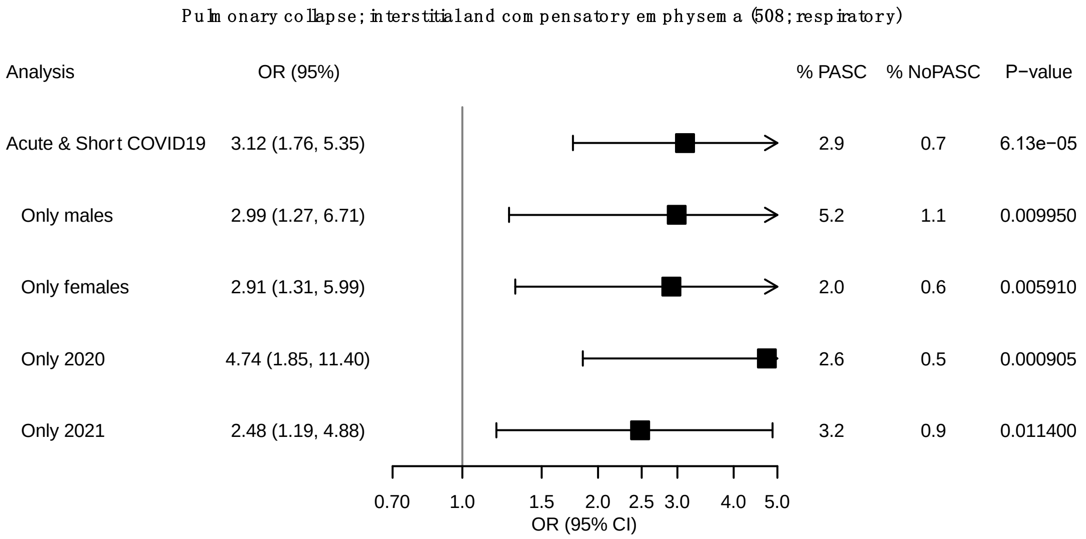
Pulmonary collapse; interstitial and compensatory emphysema (508; respiratory)
Analysis
OR (95%)
% PASC
% NoPASC
P−value
Acute & Short COVID19 3.12 (1.76, 5.35) 2.9 0.7 6.13e−05
Only males 2.99 (1.27, 6.71) 5.2 1.1 0.009950
Only females 2.91 (1.31, 5.99) 2.0 0.6 0.005910
Only 2020 4.74 (1.85, 11.40) 2.6 0.5 0.000905
Only 2021 2.48 (1.19, 4.88) 3.2 0.9 0.011400
0.70 1.0 1.5 2.0 2.5 3.0 4.0 5.0
OR (95% CI)

Z

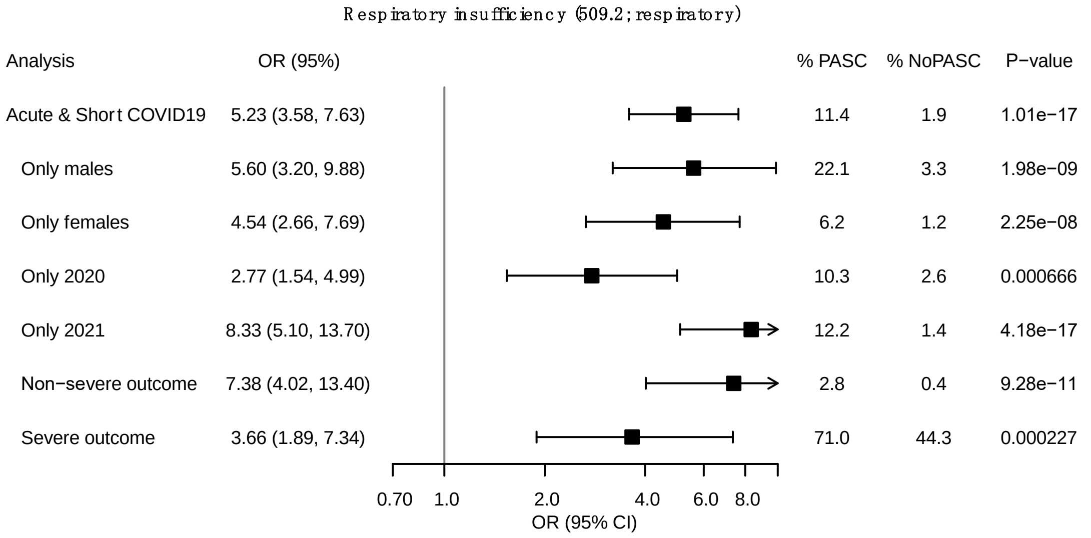
Respiratory insufficiency (509.2; respiratory)
Analysis
OR (95%)
% PASC
% NoPASC
P−value
Acute & Short COVID19 5.23 (3.58, 7.63) 11.4 1.9 1.01e−17
Only males 5.60 (3.20, 9.88) 22.1 3.3 1.98e−09
Only females 4.54 (2.66, 7.69) 6.2 1.2 2.25e−08
Only 2020 2.77 (1.54, 4.99) 10.3 2.6 0.000666
Only 2021 8.33 (5.10, 13.70) 12.2 1.4 4.18e−17
Non−severe outcome 7.38 (4.02, 13.40) 2.8 0.4 9.28e−11
Severe outcome 3.66 (1.89, 7.34) 71.0 44.3 0.000227
0.70 1.0 2.0 4.0 6.0 8.0
OR (95% CI)

AA

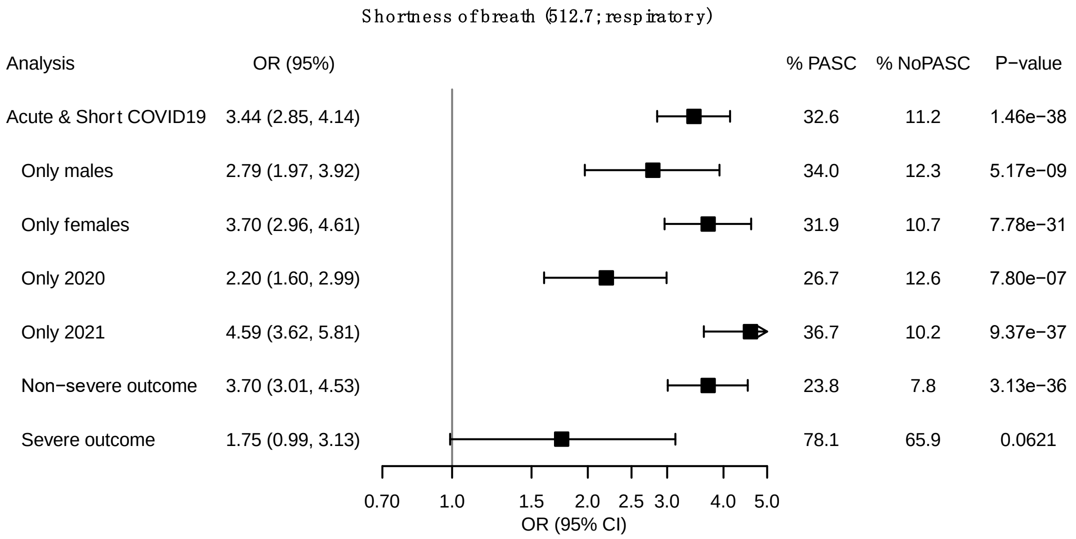
Shortness of breath (512.7; respiratory)
Analysis
OR (95%)
% PASC
% NoPASC
P−value
Acute & Short COVID19 3.44 (2.85, 4.14) 32.6 11.2 1.46e−38
Only males 2.79 (1.97, 3.92) 34.0 12.3 5.17e−09
Only females 3.70 (2.96, 4.61) 31.9 10.7 7.78e−31
Only 2020 2.20 (1.60, 2.99) 26.7 12.6 7.80e−07
Only 2021 4.59 (3.62, 5.81) 36.7 10.2 9.37e−37
Non−severe outcome 3.70 (3.01, 4.53) 23.8 7.8 3.13e−36
Severe outcome 1.75 (0.99, 3.13) 78.1 65.9 0.0621
0.70 1.0 1.5 2.0 2.5 3.0 4.0 5.0
OR (95% CI)

AB

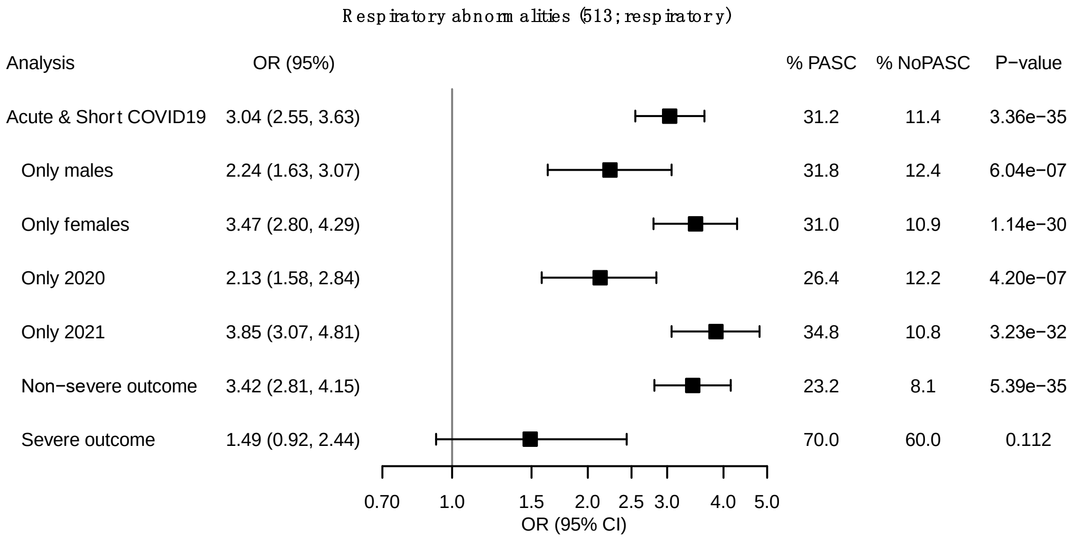
Respiratory abnormalities (513; respiratory)
Analysis
OR (95%)
% PASC
% NoPASC
P−value
Acute & Short COVID19 3.04 (2.55, 3.63) 31.2 11.4 3.36e−35
Only males 2.24 (1.63, 3.07) 31.8 12.4 6.04e−07
Only females 3.47 (2.80, 4.29) 31.0 10.9 1.14e−30
Only 2020 2.13 (1.58, 2.84) 26.4 12.2 4.20e−07
Only 2021 3.85 (3.07, 4.81) 34.8 10.8 3.23e−32
Non−severe outcome 3.42 (2.81, 4.15) 23.2 8.1 5.39e−35
Severe outcome 1.49 (0.92, 2.44) 70.0 60.0 0.112
0.70 1.0 1.5 2.0 2.5 3.0 4.0 5.0
OR (95% CI)

AC

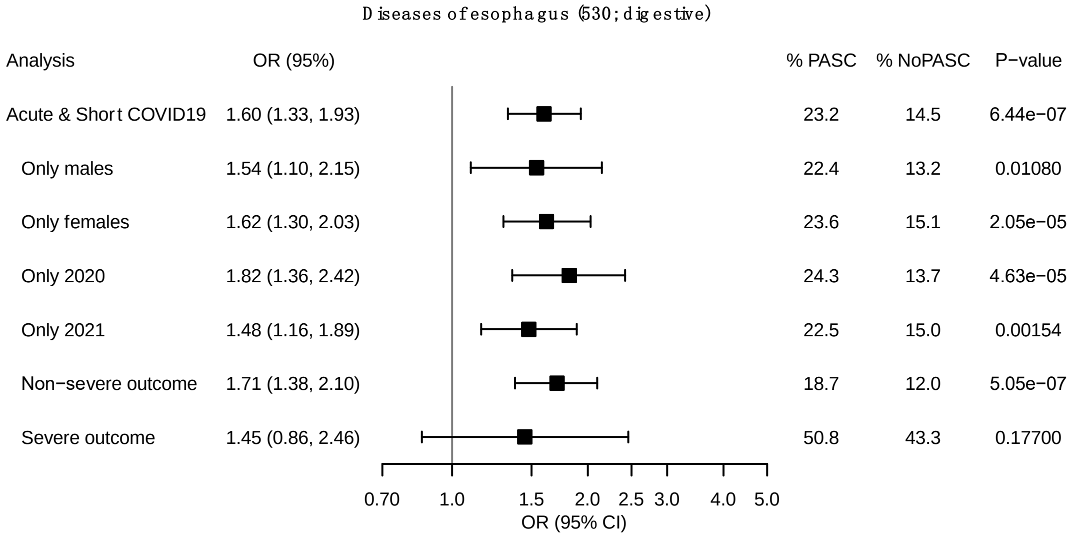
Diseases of esophagus (530; digestive)
Analysis
OR (95%)
% PASC
% NoPASC
P−value
Acute & Short COVID19
1.60 (1.33, 1.93)
23.2
14.5
6.44e−07
Only males
1.54 (1.10, 2.15)
22.4
13.2
0.01080
Only females
1.62 (1.30, 2.03)
23.6
15.1
2.05e−05
Only 2020
1.82 (1.36, 2.42)
24.3
13.7
4.63e−05
Only 2021
1.48 (1.16, 1.89)
22.5
15.0
0.00154
Non−severe outcome
1.71 (1.38, 2.10)
18.7
12.0
5.05e−07
Severe outcome
1.45 (0.86, 2.46)
50.8
43.3
0.17700
0.70
1.0
1.5
2.0
2.5
3.0
4.0
5.0
OR (95% CI)

AD

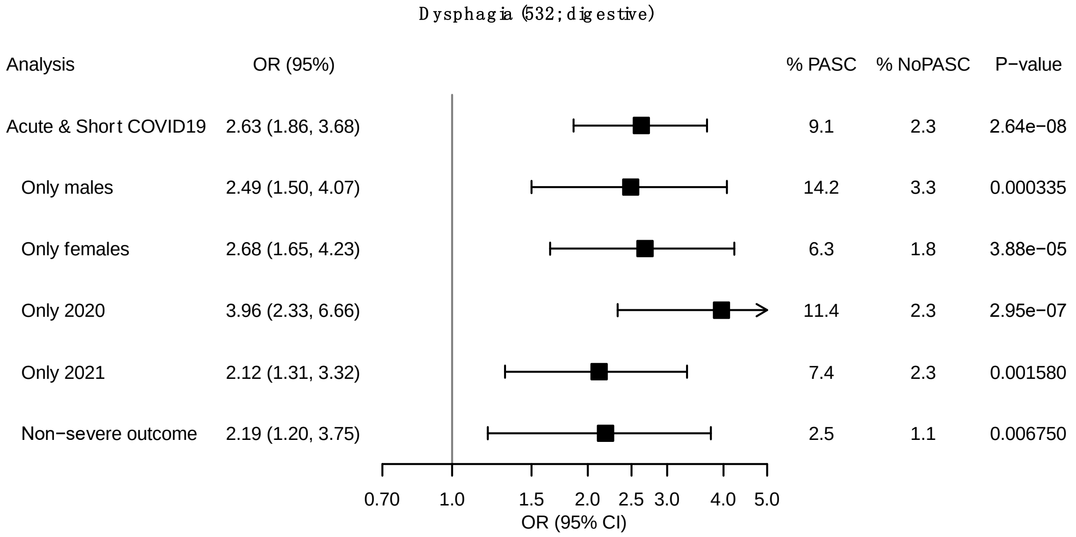
Dysphagia (532; digestive)
Analysis
OR (95%)
% PASC
% NoPASC
P−value
Acute & Short COVID19
2.63 (1.86, 3.68)
9.1
2.3
2.64e−08
Only males
2.49 (1.50, 4.07)
14.2
3.3
0.000335
Only females
2.68 (1.65, 4.23)
6.3
1.8
3.88e−05
Only 2020
3.96 (2.33, 6.66)
11.4
2.3
2.95e−07
Only 2021
2.12 (1.31, 3.32)
7.4
2.3
0.001580
Non−severe outcome
2.19 (1.20, 3.75)
2.5
1.1
0.006750
0.70
1.0
1.5
2.0
2.5
3.0
4.0
5.0
OR (95% CI)

AE

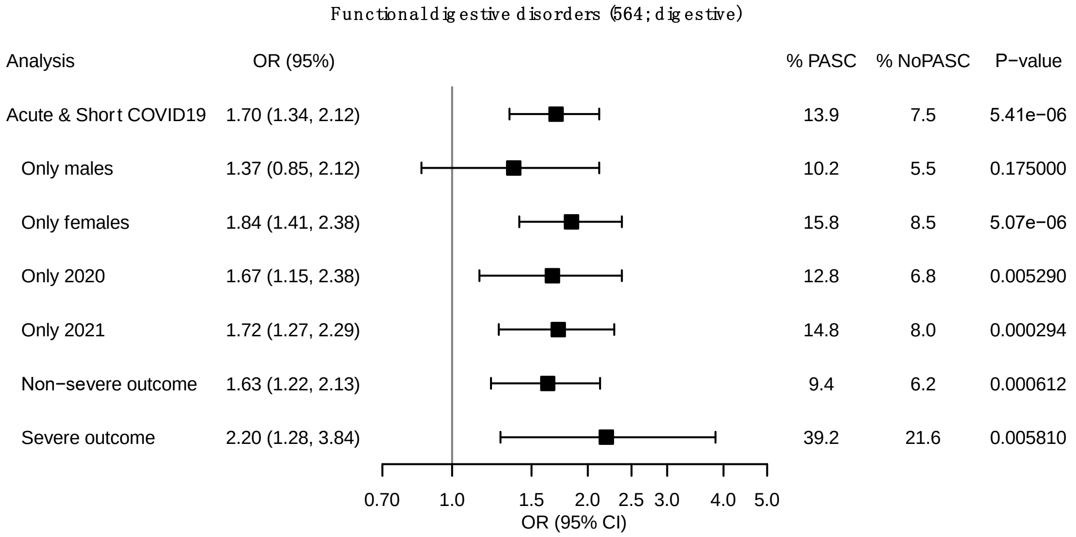
Functional digestive disorders (564; digestive)
Analysis
OR (95%)
% PASC
% NoPASC
P−value
Acute & Short COVID19
1.70 (1.34, 2.12)
13.9
7.5
5.41e−06
Only males
1.37 (0.85, 2.12)
10.2
5.5
0.175000
Only females
1.84 (1.41, 2.38)
15.8
8.5
5.07e−06
Only 2020
1.67 (1.15, 2.38)
12.8
6.8
0.005290
Only 2021
1.72 (1.27, 2.29)
14.8
8.0
0.000294
Non−severe outcome
1.63 (1.22, 2.13)
9.4
6.2
0.000612
Severe outcome
2.20 (1.28, 3.84)
39.2
21.6
0.005810
0.70
1.0
1.5
2.0
2.5
3.0
4.0
5.0
OR (95% CI)

AF

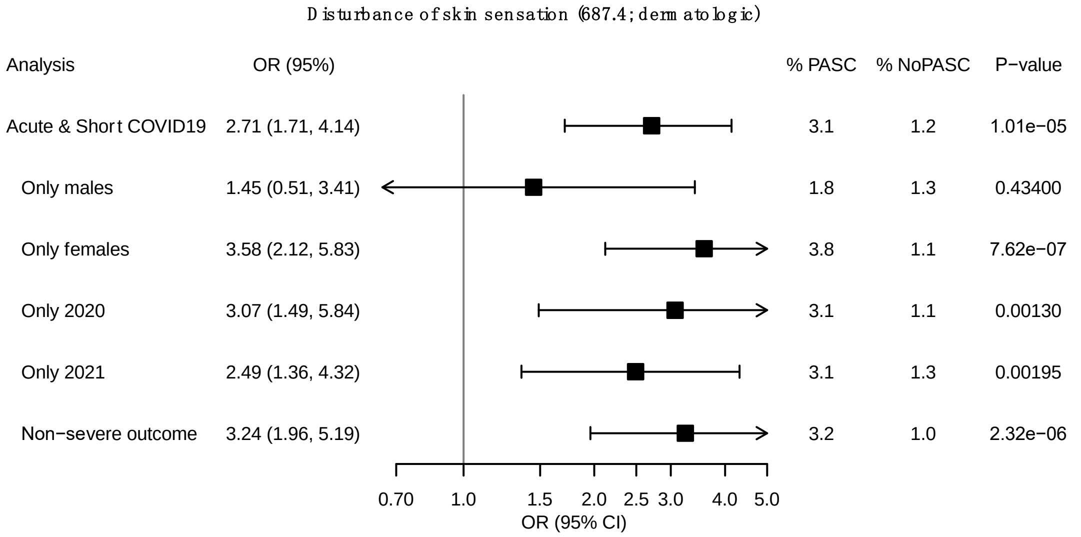
Disturbance of skin sensation (687.4; dermatologic)
Analysis
OR (95%)
% PASC
% NoPASC
P−value
Acute & Short COVID19 2.71 (1.71, 4.14) 3.1 1.2 1.01e−05
Only males 1.45 (0.51, 3.41) 1.8 1.3 0.43400
Only females 3.58 (2.12, 5.83) 3.8 1.1 7.62e−07
Only 2020 3.07 (1.49, 5.84) 3.1 1.1 0.00130
Only 2021 2.49 (1.36, 4.32) 3.1 1.3 0.00195
Non−severe outcome 3.24 (1.96, 5.19) 3.2 1.0 2.32e−06
0.70 1.0 1.5 2.0 2.5 3.0 4.0 5.0
OR (95% CI)

AG

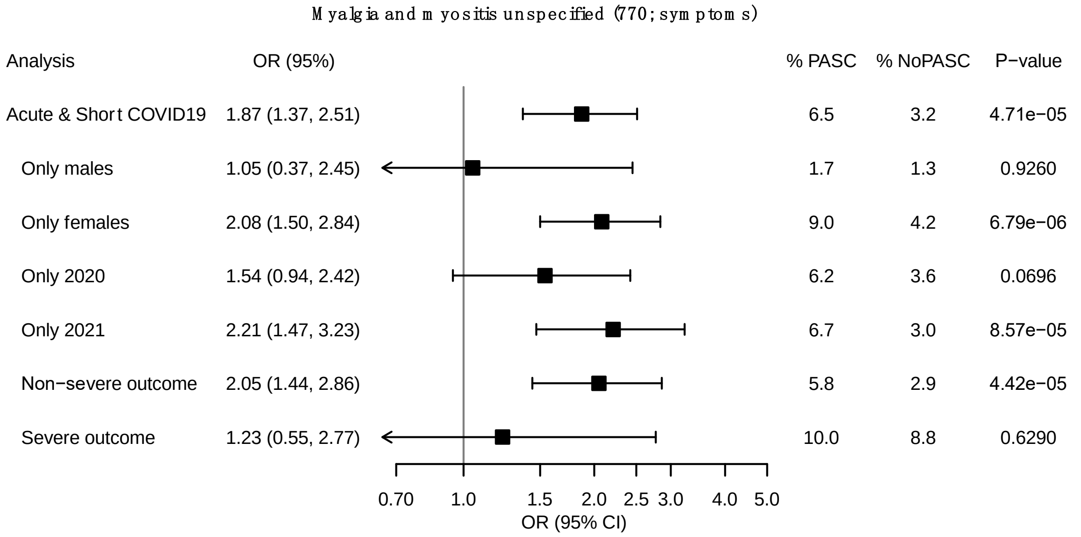
Myalgia and myositis unspecified (770; symptoms)
Analysis
OR (95%)
% PASC
% NoPASC
P−value
Acute & Short COVID19 1.87 (1.37, 2.51) 6.5 3.2 4.71e−05
Only males 1.05 (0.37, 2.45) 1.7 1.3 0.9260
Only females 2.08 (1.50, 2.84) 9.0 4.2 6.79e−06
Only 2020 1.54 (0.94, 2.42) 6.2 3.6 0.0696
Only 2021 2.21 (1.47, 3.23) 6.7 3.0 8.57e−05
Non−severe outcome 2.05 (1.44, 2.86) 5.8 2.9 4.42e−05
Severe outcome 1.23 (0.55, 2.77) 10.0 8.8 0.6290
0.70 1.0 1.5 2.0 2.5 3.0 4.0 5.0
OR (95% CI)

AH

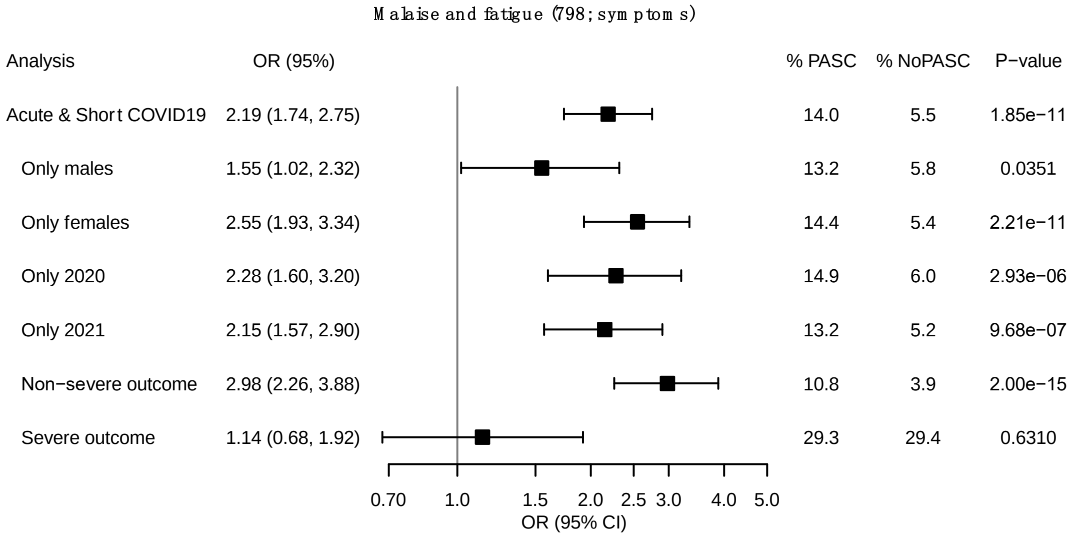
Malaise and fatigue (798; symptoms)
Analysis
OR (95%)
% PASC
% NoPASC
P−value
Acute & Short COVID19 2.19 (1.74, 2.75) 14.0 5.5 1.85e−11
Only males 1.55 (1.02, 2.32) 13.2 5.8 0.0351
Only females 2.55 (1.93, 3.34) 14.4 5.4 2.21e−11
Only 2020 2.28 (1.60, 3.20) 14.9 6.0 2.93e−06
Only 2021 2.15 (1.57, 2.90) 13.2 5.2 9.68e−07
Non−severe outcome 2.98 (2.26, 3.88) 10.8 3.9 2.00e−15
Severe outcome 1.14 (0.68, 1.92) 29.3 29.4 0.6310
0.70 1.0 1.5 2.0 2.5 3.0 4.0 5.0
OR (95% CI)

AI

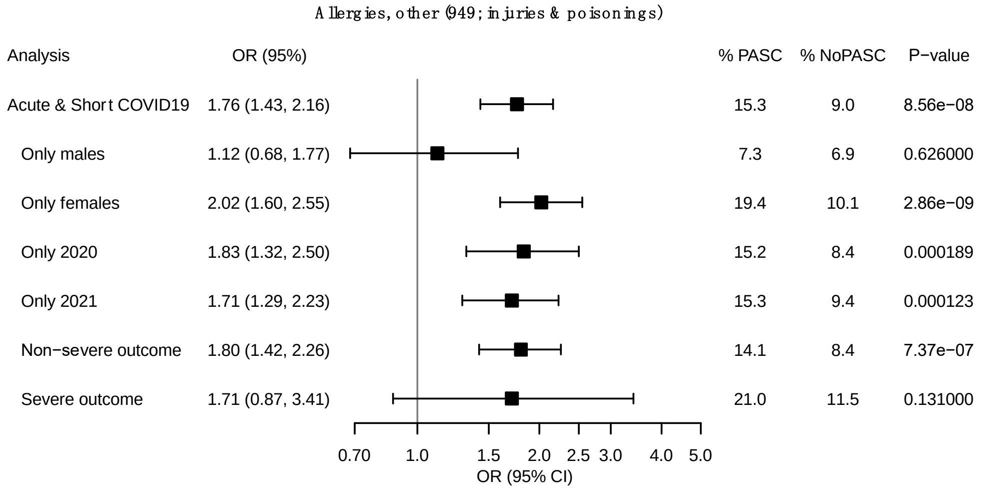

AJ

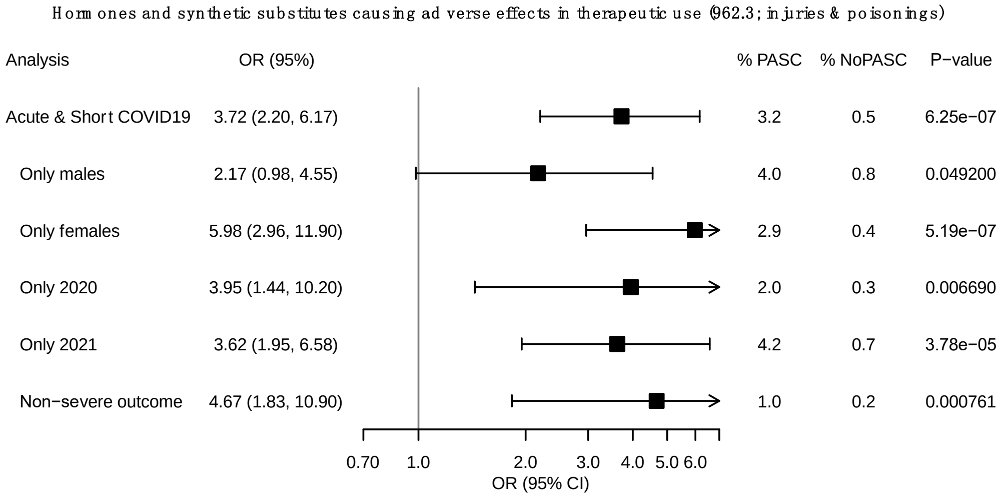

AK

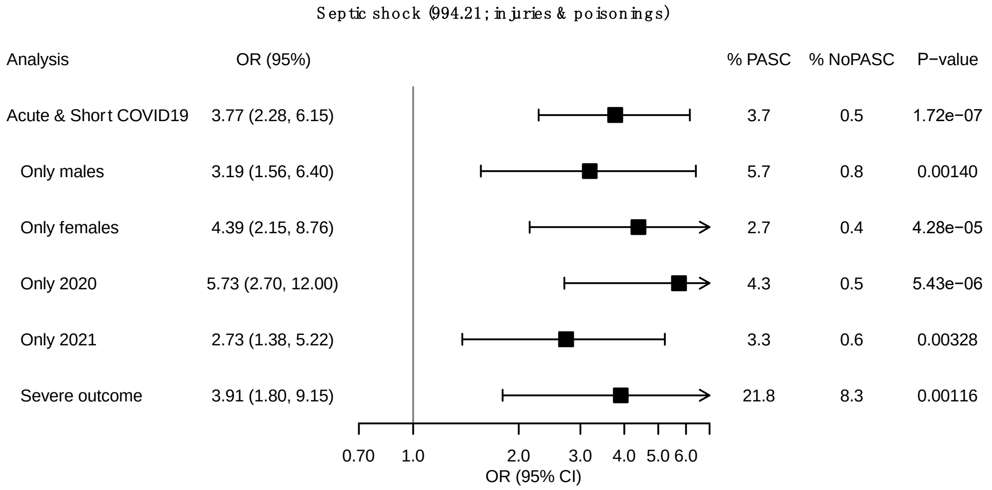

Figure S4. A-Z, AA-AK. Forest plots of the Acute & short COVID-19 Sensitivity analyses. Effect sizes and phecode frequencies in cases (PASC) and controls (No PASC) of significantly associated PheCodes are shown. Acute & short COVID-19: main analysis, only males, only females, COVID-19 positive in 2020, COVID-19 positive in 2021, non-severe COVID-19 outcome only, and severe COVID-

19 outcome. Of significantly associated parent/child phecodes only the phecode with the stronger association signal is shown. Sample sizes of each analysis can be found in **Table S5**.

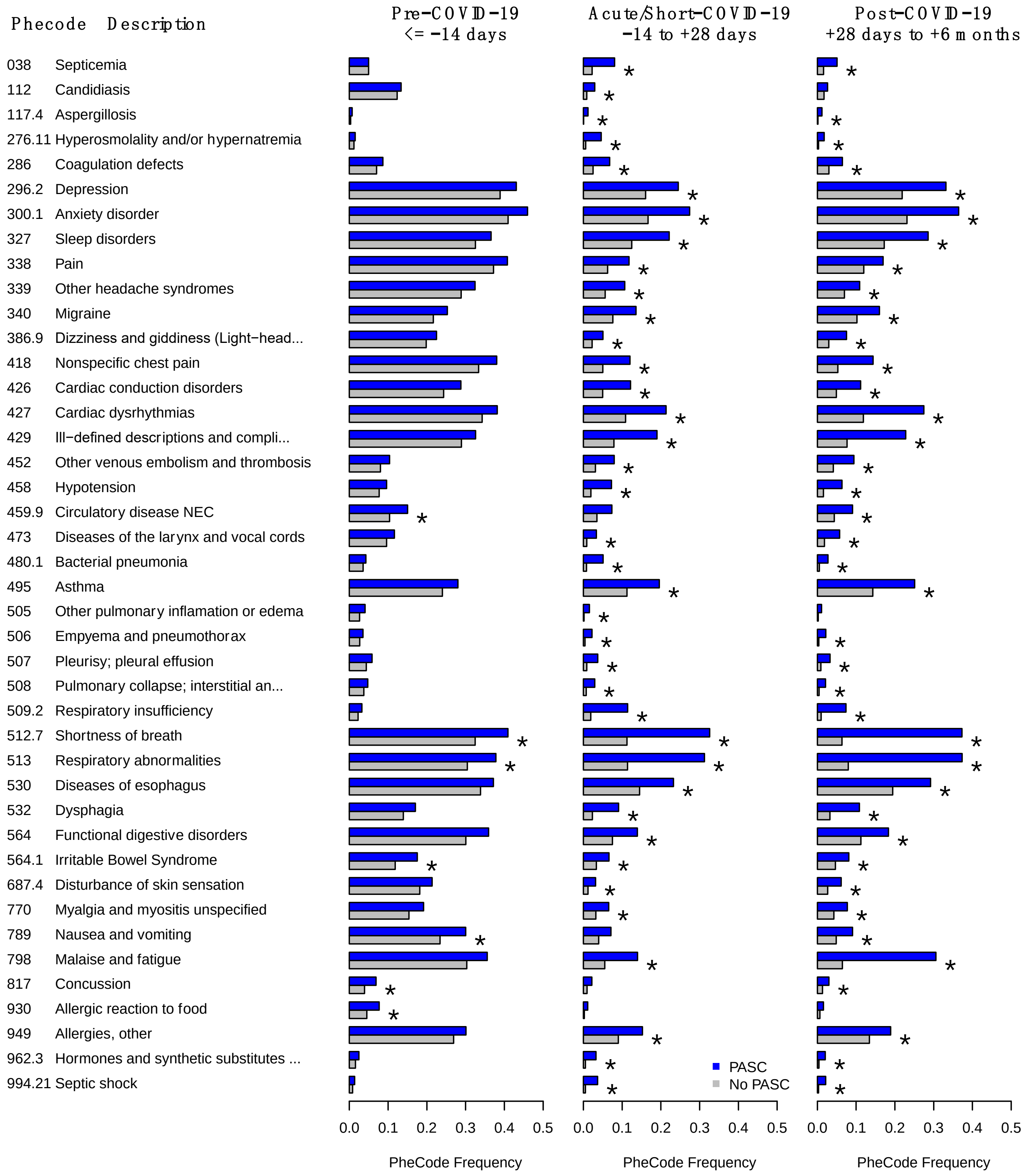

Figure S5. Comparison of PheCode prevalence during pre-COVID-19, acute/short-COVID-19, and post-COVID-19 periods in cases and controls. Only PheCodes that were significantly enriched among PASC patients in the pre-COVID-19 and/or acute/short-COVID-19 periods are shown. Of parent/child PheCodes only the phecode with the stronger association signal is shown. PheCodes that were

phenome-wide significant in the corresponding PheWAS are indicated with an asterisk. Phenome-wide significant enriched PheCodes are indicated by an asterisk.

Table S1. PASC Problem list.

Problem list description	Mapped to ICD10 Code
Chronic post-COVID-19 syndrome	B94.8
Shortness of breath with exposure to COVID-19 virus	R06.02
COVID-19 long hauler	U09.9
COVID-19 long hauler manifesting chronic anxiety	U09.9
COVID-19 long hauler manifesting chronic concentration deficit	U09.9
COVID-19 long hauler manifesting chronic cough	U09.9
COVID-19 long hauler manifesting chronic dyspnea	U09.9
COVID-19 long hauler manifesting chronic fatigue	U09.9
COVID-19 long hauler manifesting chronic headache	U09.9
COVID-19 long hauler manifesting chronic joint pain	U09.9
COVID-19 long hauler manifesting chronic loss of smell	U09.9
COVID-19 long hauler manifesting chronic loss of smell and taste	U09.9
COVID-19 long hauler manifesting chronic loss of taste	U09.9
COVID-19 long hauler manifesting chronic muscle pain	U09.9
COVID-19 long hauler manifesting chronic neurologic symptoms	U09.9
COVID-19 long hauler manifesting chronic palpitations	U09.9
Long COVID	U09.9
Multiple persistent symptoms after COVID-19	U09.9
Persistent dyspnea after COVID-19	U09.9
Persistent fatigue after COVID-19	U09.9
Persistent neurologic symptoms after COVID-19	U09.9
Persistent shortness of breath after COVID-19	U09.9
Post covid-19 condition, unspecified	U09.9
Post-acute COVID-19 syndrome	U09.9
Post-acute sequelae of COVID-19 (PASC)	U09.9
Post-COVID chronic anxiety	U09.9
Post-COVID chronic concentration deficit	U09.9
Post-COVID chronic cough	U09.9
Post-COVID chronic dyspnea	U09.9
Post-COVID chronic fatigue	U09.9
Post-COVID chronic headache	U09.9
Post-COVID chronic joint pain	U09.9
Post-COVID chronic loss of smell	U09.9
Post-COVID chronic loss of smell and taste	U09.9
Post-COVID chronic loss of taste	U09.9
Post-COVID chronic muscle pain	U09.9
Post-COVID chronic neurologic symptoms	U09.9
Post-COVID chronic palpitations	U09.9
Post-COVID chronic shortness of breath	U09.9
Post-COVID syndrome	U09.9
Post-COVID-19 condition	U09.9
Post-COVID-19 syndrome	U09.9
Post-COVID-19 syndrome manifesting as chronic anxiety	U09.9
Post-COVID-19 syndrome manifesting as chronic concentration deficit	U09.9
Post-COVID-19 syndrome manifesting as chronic cough	U09.9
Post-COVID-19 syndrome manifesting as chronic dyspnea	U09.9
Post-COVID-19 syndrome manifesting as chronic fatigue	U09.9
Post-COVID-19 syndrome manifesting as chronic headache	U09.9
Post-COVID-19 syndrome manifesting as chronic joint pain	U09.9
Post-COVID-19 syndrome manifesting as chronic loss of smell	U09.9
Post-COVID-19 syndrome manifesting as chronic loss of smell and taste	U09.9
Post-COVID-19 syndrome manifesting as chronic loss of taste	U09.9
Post-COVID-19 syndrome manifesting as chronic muscle pain	U09.9
Post-COVID-19 syndrome manifesting as chronic neurologic symptoms	U09.9
Post-COVID-19 syndrome manifesting as chronic palpitations	U09.9

Post-COVID-19 syndrome manifesting as chronic shortness of breath	U09.9
COVID-19 long hauler manifesting chronic decreased mobility and endurance	Z74.09
Post-COVID chronic decreased mobility and endurance	Z74.09
Post-COVID-19 syndrome manifesting as chronic decreased mobility and endurance	Z74.09

Table S2. PASC symptom and concurrent symptom mapping.

PheCode	PheCode Description	PSL Key Words	ICD10 Diagnosis mapped to PSL	Symptom described in Chen et al 2022
260.6	Anorexia	Appetite	R63.0	Appetite / Eating disorder
292	Neurological disorders	Neurologic or cognitive deficit	R41	Memory problems / Concentration / Confusion / Brain fog
296.2	Depression	Depression	F32	Depression
300.1	Anxiety disorder	Anxiety	F41	Anxiety
327	Sleep disorders	Sleep apnea	G47	Sleep problems
338.2	Chronic pain	Chronic pain	G89.2	n/a
339	Any headache syndromes	Headache	R51	Headache
340	Migraine	Migraine	G43	n/a
350.6	Disturbances of sensation of smell and taste	Smell and taste	R43.8	Smell or Taste
386.9	Dizziness and giddiness (Light-headedness and vertigo)	Dizziness	R42	Dizziness
418	Nonspecific chest pain	Chest pain	R07	Chest pain
427.7	Tachycardia NOS	Tachycardia	R00	Tachycardia
427.9	Palpitations	Palpitations	R00	n/a
465.2	Acute pharyngitis	Sore throat	J02.9	Sore throat
473.4	Voice disturbance	Dysphonia	R49.0	n/a
495	Asthma	Asthma	J45	n/a
496	Chronic airway obstruction	COPD	J44	n/a
512.7	Shortness of breath	Dyspnea	R06.0	Dyspnea
512.8	Cough	Cough	R05	Cough
561	Symptoms involving digestive system	Diarrhea	R19.7	Diarrhea
687.1	Rash and other nonspecific skin eruption	Rash	R21	n/a
704.1	Alopecia	Hair loss	L65	Hair loss
745	Pain in joint	Joint pain	M25.5	Joint pain
760	Back pain	Back pain	M54	n/a
770	Myalgia and myositis unspecified	Myalgia	M79.1	Myalgia
772.3	Muscle weakness	Muscle weakness	M62.81	n/a
783	Fever of unknown origin	Fever	R50	Fever
785	Abdominal pain	Abdominal pain	R10	Abdominal pain
798	Malaise and fatigue	Fatigue	R53	Fatigue

Table S3. Covariate summary and missingness in the unmatched and matched cohort (see **Figure 2**).

Standard Set of Covariates			**Missingness, n (%)**	
**Covariate**	**Data Type**		**Unmatched cohort n = 63,675**	**Matched cohort n = 18,929**
Age at positive COVID-19 test/diagnosis	Continuous		0 (0)	0 (0)[e]
Gender	Binary		5 (0)	0 (0)[e]
Self-reported Race / Ethnicity [a]	Categorical		0 (0)	0 (0)[e]
Elixhauser Score (AHRQ)	Continuous		0 (0)	0 (0)
NDI	Categorical (quartiles)		5,793 (9.1)	1,335 (7.1)
Population density	Categorical (quartiles)		5,793 (9.1)	1,335 (7.1)
Health Care Worker Status	Binary		0 (0)[f]	0 (0)[f]
Severity [b]	Binary		0 (0)[f]	0 (0)[f]
Vaccination status at time of test [c]	Categorical		0 (0)[f]	0 (0)[f]
Analysis dependent Covariates				
**Analysis**	**Covariate**	**Data Type**	**Missingness, n (%)**	
Pre-Pandemic PheWAS	Pre-Pandemic Years in EHR	Continuous	1,360 (2.1)	99 (0.5)
Pre-COVID-19 PheWAS	Pre-Test Years in EHR	Continuous	0 (0)	0 (0)[e]
Acute & Short COVID-19 PheWAS	Post-COVID-19-Test Years in EHR	Continuous	0 (0)	0 (0)[e]
Post-COVID-19 Symptom PheWAS	Post-COVID-19-Test Years in EHR	Continuous	0 (0)	0 (0)[e]

[a] Self-reported Race / Ethnicity factor levels: Caucasian/Non-Hispanic, African American/Non-Hispanic, Other Race or Ethnicity, and Unknown Race or Ethnicity. [b] mild: asymptomatic/non-hospitalized; severe: hospitalized, ICU stay or deceased. [c] unvaccinated, partially vaccinated, fully vaccinated, boosted. [e] complete data was requirement for matching. [f] based on available EHR documentation (“documented” versus “not documented or unknown”); actual missingness unknown.

Table S4. Main and sensitivity PheWAS. Each analysis only includes individual who were COVID-19 positive for the first time before 2022.

**Analysis**	**Phenome Description (relative to index date)**	**n PASC**	**n No PASC (Matched)**	**n PheCodes***
**Main Analysis**	Pre-existing conditions / pre-COVID-19 symptoms (before -14 days)	1,212	11,919	1,405
**Sensitivity Analyses**	Females only	793	7,882	1,237
	Males only	419	4,037	928
	1. Positive in 2020	381	3,671	1,013
	1. Positive in 2021	831	8,248	1,285
	Asymptomatic / mild COVID-19 outcome	1,009	9,393	1,273
	Severe COVID-19 outcome	203	204	944
	Max 2 years before COVID-19 test	1,212	11,919	1,061
	Pre-existing conditions before pandemic (before 2020)	1,085	10,533	1,347
**Main Analysis**	Acute / short COVID-19 period symptoms (between -14 and + 28 days)	874	8,671	664
**Sensitivity Analyses**	Females only	578	5,766	510
	Males only	296	2,905	365
	1. Positive in 2020	368	3,623	397
	2. Positive in 2021	506	5,048	504
	Asymptomatic / Mild COVID-19	724	6,799	454
	Severe COVID-19	150	160	419
**Main Analysis**	Post-COVID-19 symptoms (between +28 days and +6 months)	1,256	12,492	961

* PheCode prevalence >= 5 among individuals with and without PASC (complete case analysis).

Table S5. Concurrent diagnoses on day of the first PASC diagnosis. Only diagnoses that matched the 24 listed categories are shown.

Symptoms	n	% with Unspecified PASC (n = 1,724)	% Without Unspecified PASC (n = 1,362)
Shortness of breath	469	27.2	34.4
Anxiety disorder	417	24.2	30.6
Malaise and fatigue	388	22.5	28.5
Depression	370	21.5	27.2
Sleep disorders	346	20.1	25.4
Asthma	321	18.6	23.6
Other headache syndromes	291	16.9	21.4
Migraine	188	10.9	13.8
Cough	177	10.3	13.0
Pain in joint	172	10.0	12.6
Back pain	158	9.2	11.6
Neurological disorders	155	9.0	11.4
Nonspecific chest pain	143	8.3	10.5
Chronic pain	132	7.7	9.7
Tachycardia NOS	114	6.6	8.4
Myalgia and myositis unspecified	115	6.7	8.4
Chronic airway obstruction	101	5.9	7.4
Symptoms involving digestive system	100	5.8	7.3
Abdominal pain	91	5.3	6.7
Palpitations	66	3.8	4.8
Rash and other nonspecific skin eruption	65	3.8	4.8
Dizziness and giddiness (Light-headedness and vertigo)	64	3.7	4.7
Fever of unknown origin	46	2.7	3.4
Disturbances of sensation of smell and taste	44	2.6	3.2
Voice disturbance	23	1.3	1.7
Muscle weakness	15	0.9	1.1
Acute pharyngitis	12	0.7	0.9
Anorexia	11	0.6	0.8
Alopecia	8	0.5	0.6
Unspecified PASC	362	21.0	0.0

Table S6. Enrichment 29 known PASC symptoms among post-COVID-19 diagnoses (observed between +28 days and 6 months after being COVID-19 positive) in PASC cases compared to "No PASC" controls.

PheCode	PheCode Description	PheCode Category	PheCode Frequeny [PASC]	PheCode Frequency [No PASC]	OR (95% CI)	P-value	Significance Level
512.7	Shortness of breath	respiratory	425 / 1139 (37.3)	744 / 11752 (6.3)	9.03 (7.77, 10.5)	2.94E-181	phenome-wide
798	Malaise and fatigue	symptoms	384 / 1256 (30.6)	803 / 12492 (6.4)	6.17 (5.33, 7.14)	2.32E-132	phenome-wide
512.8	Cough	respiratory	136 / 850 (16.0)	457 / 11465 (4.0)	4.39 (3.54, 5.41)	1.61E-42	phenome-wide
292	Neurological disorders	mental disorders	160 / 1228 (13.0)	439 / 12274 (3.6)	3.57 (2.91, 4.35)	8.85E-36	phenome-wide
427.7	Tachycardia NOS	circulatory system	118 / 1003 (11.8)	286 / 11112 (2.6)	4.39 (3.45, 5.56)	5.58E-34	phenome-wide
418	Nonspecific chest pain	circulatory system	180 / 1253 (14.4)	660 / 12487 (5.3)	2.87 (2.39, 3.44)	2.98E-30	phenome-wide
427.9	Palpitations	circulatory system	97 / 982 (9.9)	306 / 11132 (2.7)	3.88 (3.03, 4.92)	2.02E-28	phenome-wide
300.1	Anxiety disorder	mental disorders	406 / 1114 (36.4)	2628 / 11368 (23.1)	1.95 (1.70, 2.23)	4.55E-22	phenome-wide
350.6	Disturbances of sensation of smell and taste	neurological	< 50 / 1172 (3.0)	< 50 / 12091 (0.4)	7.69 (4.93, 11.9)	4.51E-21	phenome-wide
327	Sleep disorders	neurological	358 / 1253 (28.6)	2152 / 12478 (17.2)	1.92 (1.67, 2.20)	1.11E-20	phenome-wide
495	Asthma	respiratory	300 / 1196 (25.1)	1742 / 12171 (14.3)	1.98 (1.72, 2.29)	1.36E-20	phenome-wide
386.9	Dizziness and giddiness (Light-headedness and vertigo)	sense organs	94 / 1252 (7.5)	367 / 12434 (3.0)	2.68 (2.10, 3.39)	3.65E-16	phenome-wide
296.2	Depression	mental disorders	351 / 1059 (33.1)	2448 / 11188 (21.9)	1.79 (1.55, 2.07)	8.83E-16	phenome-wide
783	Fever of unknown origin	symptoms	73 / 1256 (5.8)	196 / 12492 (1.6)	3.15 (2.34, 4.19)	6.41E-15	phenome-wide
473.4	Voice disturbance	respiratory	< 50 / 958 (4.3)	113 / 10361 (1.1)	3.43 (2.33, 4.94)	5.86E-11	phenome-wide
340	Migraine	neurological	190 / 1187 (16.0)	1224 / 12007 (10.2)	1.75 (1.47, 2.08)	1.74E-10	phenome-wide
770	Myalgia and myositis unspecified	symptoms	97 / 1256 (7.7)	532 / 12492 (4.3)	1.87 (1.48, 2.35)	9.22E-08	phenome-wide
785	Abdominal pain	symptoms	159 / 1256 (12.7)	1003 / 12492 (8.0)	1.61 (1.34, 1.93)	2.80E-07	phenome-wide
760	Back pain	symptoms	225 / 1256 (17.9)	1555 / 12492 (12.4)	1.51 (1.28, 1.76)	4.45E-07	phenome-wide
496	Chronic airway obstruction	respiratory	92 / 988 (9.3)	507 / 10936 (4.6)	1.93 (1.49, 2.49)	4.92E-07	phenome-wide
745	Pain in joint	musculoskeletal	219 / 1256 (17.4)	1537 / 12492 (12.3)	1.51 (1.28, 1.77)	5.62E-07	phenome-wide
772.3	Muscle weakness	symptoms	< 50 / 1221 (2.9)	103 / 12252 (0.8)	2.67 (1.76, 3.95)	1.11E-06	phenome-wide
704.1	Alopecia	dermatologic	< 50 / 1238 (3.5)	177 / 12339 (1.4)	2.31 (1.62, 3.22)	1.39E-06	phenome-wide
339	Other headache syndromes	neurological	122 / 1119 (10.9)	808 / 11591 (7.0)	1.65 (1.34, 2.03)	2.21E-06	phenome-wide
465.2	Acute pharyngitis	respiratory	< 50 / 1221 (2.8)	162 / 12232 (1.3)	2.27 (1.53, 3.28)	1.66E-05	phenome-wide
338.2	Chronic pain	neurological	164 / 1207 (13.6)	1122 / 12121 (9.3)	1.49 (1.24, 1.78)	1.87E-05	phenome-wide
687.1	Rash and other nonspecific skin eruption	dermatologic	78 / 1183 (6.6)	472 / 12148 (3.9)	1.71 (1.32, 2.19)	2.71E-05	phenome-wide
561	Symptoms involving digestive system	digestive	< 50 / 981 (1.9)	139 / 10676 (1.3)	1.56 (0.93, 2.47)	0.069	n.s.
260.6	Anorexia	endocrine/metabolic	< 50 / 1047 (0.8)	< 50 / 11384 (0.4)	1.81 (0.79, 3.70)	0.106	n.s.

Notes: phenome-wide: P < 0.05/961 tested PheCodes (P < 5.1E-05); n.s.: not significant.

Table S7. PheRS Evaluation in the testing data (COVID-19 positive in 2022). PheRS1* was based on phecodes that reached P < 1-E3 in the PheWAS with the pre-COVID-19 training data (1,256 cases and 11,674 controls; COVID-19 positive in 2020/2021), while PheRS2* was based on phecodes that reached P < 1-E3 in the PheWAS with the acute & short COVID-19 training data (874 cases and 8,144 controls; COVID-19 positive in 2020/2021 & at least 28 days between first COVID-19 and first PASC diagnosis). Underlying weights can be found in **File S1J and Table S8**.

Predictor	Testing Data		AAUC [a] 95% CI	Pseudo-R^2 [b]	Brier Score
	n Cases	n Controls			
PheRS1*	349	3248	0.545 (0.514, 0.576)	n/a [c]	n/a [c]
PheRS1*			0.551 (0.495, 0.611)	0.0086	0.0857
PheRS2*	123	1154	0.595 (0.540, 0.653)	0.0439	0.0830
PheRS1 & PheRS2*			0.601 (0.548, 0.658)	0.0435	0.0831

[a] Adjusted for age at index date, gender, race/ethnicity, Elixhauser Score, population density, NDI, health care worker status, vaccination status, pre-test years in EHR, and severity. [b] Nagelkerke [Cragg and Uhler]).[c] not applicable, only useful in evaluating multiple models predicting the same outcome on the same dataset.

Table S8. Weights for combining PheRS1 and PheRS2 or PheRS1* and PheRS2*. PheRS were standardized (mean =0 and sd = 1) before calculating the weights and before calculating the combined PheRSs.

PheRS Combination	Predictor	Weight*
PheRS1 & PheRS2 Combined	PheRS1	0.08289
	PheRS2	0.52371
PheRS1* & PheRS2* Combined	PheRS1*	0.08267
	PheRS2*	0.51564

Disclaimer/Publisher's Note: The statements, opinions and data contained in all publications are solely those of the individual author(s) and contributor(s) and not of MDPI and/or the editor(s). MDPI and/or the editor(s) disclaim responsibility for any injury to people or property resulting from any ideas, methods, instructions or products referred to in the content.
